# Polymer-Sensitized Hybrid Design Strategy for High-Efficiency Blue Hyperfluorescent OLEDs

**DOI:** 10.1126/sciadv.aee0158

**Published:** 2026-04-10

**Authors:** Junwon Jeon, Hyung Jin Cheon, Joo Yoon Woo, Hyuk Bin Kwon, Min-Ho Park, Yun-Hi Kim, Tae-Hee Han

**Affiliations:** ^1^ Department of Display Science and Engineering, Hanyang University, 222 Wangsimni-ro, Seongdong-gu, Seoul 04763, Republic of Korea.; ^2^ Department of Chemistry and RIMA, Gyeongsang National University, Jinju 52828, Republic of Korea.; ^3^ Division of Materials Science and Engineering, Hanyang University, 222 Wangsimni-ro, Seongdong-gu, Seoul 04763, Republic of Korea.; ^4^ Department of Materials Science and Engineering, Soongsil University, 369 Sangdo-ro, Dongjak-gu, Seoul 06978, Republic of Korea.

## Abstract

Achieving efficient and color–pure-blue emission in solution-processed organic light-emitting diodes (SOLEDs) remains a challenge due to poor triplet utilization and interfacial energy loss. We report high-efficiency blue hyperfluorescent (HF) SOLEDs that use a polymer/small-molecule hybrid emitting layer composed of a polymeric thermally activated delayed fluorescence (TADF) sensitizer, poly(10-(3-(4-(8-phenyloctyl)phenyl)-5,9-dioxa-13b-boranaphtho[3,2,1-de]anthracen-7-yl)-10H-spiro[acridine-9,9′-fluorene]), and a narrowband multiresonance TADF emitter. The polymer sensitizer enables efficient triplet harvesting and rapid reverse intersystem crossing, suppresses aggregation, and ensures effective energy transfer to the terminal emitter. In addition, self-organized polymeric hole injection layers are introduced to increase hole injection and suppress exciton loss at the interface. As a result, blue SOLEDs achieved a high external quantum efficiency of 32.7%, which is the highest reported to date for polymer-based TADF or HF OLEDs. This study demonstrates a polymer-sensitized blue HF OLED and offers a generalizable strategy for production of high-efficiency SOLED platforms.

## Teaser

A hybrid emitter design using polymer sensitization achieves highly efficient and pure-blue emission from printable OLEDs.

## INTRODUCTION

Organic light-emitting diodes (OLEDs) have become essential in modern optoelectronics, which offer high efficiency, mechanical flexibility, and exceptional color quality for displays and lighting (*1*–*5*). In particular, solution-processed OLEDs (SOLEDs) have potential for cost-effective, large-area, and scalable fabrication and therefore constitute promising alternatives to vacuum-deposited devices (*6*, *7*).

Hyperfluorescence (HF) combines a thermally activated delayed fluorescence (TADF) sensitizer with a fluorescence terminal emitter. HF is a promising OLED configuration to achieve narrowband and high-efficiency light emission (*8*–*17*). In this architecture, the TADF sensitizer efficiently harvests both singlet and triplet excitons by reverse intersystem crossing (RISC), followed by Förster resonance energy transfer (FRET) to the terminal emitter; this process helps reduce accumulation of triplet excitons on the emitter and thereby mitigates exciton annihilation and increases device efficiency (*18*). In addition, the use of a multiresonance TADF (MR-TADF) emitter as the terminal emitter increases singlet exciton emission and enables ultranarrow emission bandwidth and high color purity (*12*, *19*).

However, using HF to achieve efficient pure-blue emission in SOLEDs is a challenging task. Solution-processed emitting layers (EMLs) often undergo phase separation, have poor morphological stability, and develop charge imbalance due to their simple architecture and wet-coating nature (*6*, *20*). These problems can increase when using MR-TADF emitters, which have low triplet-harvesting capability and are prone to aggregation-induced quenching and exciton annihilation in solution (*21*, *22*). In addition, the simplified device architecture of SOLEDs often leads to substantial energy-level mismatches between the hole injection layer (HIL) and the EML; this mismatch leads to inefficient hole injection and exacerbates charge imbalance. These challenges limit the achievable luminous efficiency and spectral purity in blue-emitting HF OLEDs fabricated using solution techniques.

Polymeric TADF sensitizers can offer several attractive features, such as excellent film-forming ability, high thermal stability, and suppressed molecular aggregation (*23*, *24*). Furthermore, incorporation of flexible side chains can minimize aggregation-induced nonradiative exciton annihilation and enable uniform dispersion of terminal emitters within the host matrix (*25*, *26*). Therefore, developing polymer-sensitized HF systems that are compatible with MR-TADF emitters and maintain both spectral integrity and device efficiency remains particularly important for blue-emitting OLEDs.

Realizing high-efficiency devices that use blue polymeric TADF emitters is a major challenge (*27*, *28*). In the predominantly studied TADF polymers that have a π-conjugated backbone, blue-emission properties are constrained because the extended backbone often lowers the singlet energy level (S_1_) and induces energy back transfer, which causes red-shifted emission and nonradiative losses that hinder efficient blue emission (*29*).

To overcome these limitations of blue-emitting HF SOLEDs, we adopted a dual design strategy that combines engineering at both emitter and interface levels. A TADF polymer–based hybrid EML was constructed by incorporating a blue-emitting TADF polymer and a small-molecule MR-TADF narrowband emitter, which enables efficient triplet harvesting, suppresses molecular aggregation, and increases FRET within a morphologically stable EML matrix. Spontaneously formed self-organized polymeric HILs were also introduced to facilitate energy level alignment, improve hole injection, and suppress interfacial exciton quenching. This integrated approach yielded blue-emitting HF SOLEDs that deliver both high efficiency and spectrally pure-blue emission.

## RESULTS

### Synthesis and characterization of TADF polymer

**Fig. 1.**
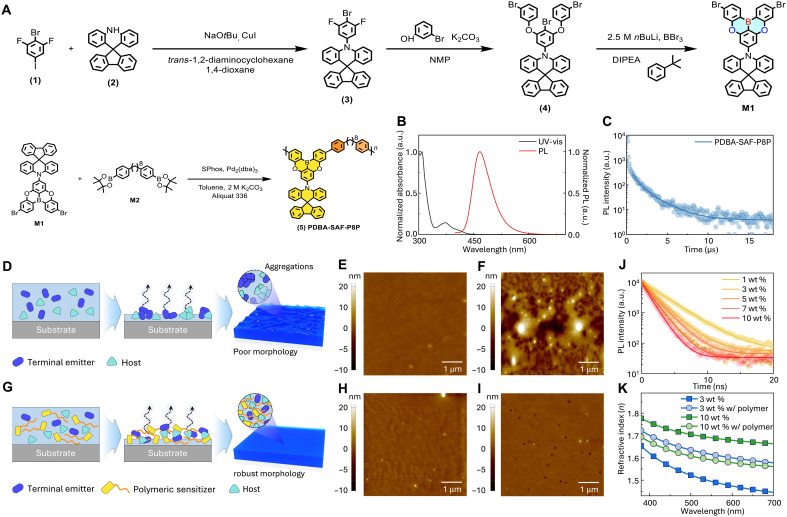
Synthesis and photophysical properties of TADF polymer. (**A**) Synthesis of PDBA-SAF-P8P. (**B**) Ultraviolet-visible (UV-vis) absorption and photoluminescence (PL) spectra of PDBA-SAF-P8P in toluene (10^–5^ M). a.u., arbitrary units. (**C**) Time-resolved PL (TRPL) spectra of PDBA-SAF-P8P in neat film at room temperature. (**D**) Schematic illustrations of film formation by solution processing with planar MR-TADF emitter molecules within small molecular host matrix. Atomic force microscopy (AFM) images of solution-processed films (mCP:DBFPO:ν-DABNA), with emitter weight ratios of (**E**) 3 wt % and (**F**) 10 wt %. (**G**) Schematic illustrations of film formation by solution processing, with MR-TADF molecules uniformly dispersed within the host matrix using a polymer sensitizer. AFM images of solution-processed films (mCP:DBFPO:PDBA-SAF-P8P:ν-DABNA), with 33 wt % polymer sensitizer and emitter weight ratios of (**H**) 3 wt % and (**I**) 10 wt %. (**J**) TRPL spectra of solution-processed films (mCP:DBFPO:ν-DABNA) with various concentrations of MR-TADF emitter. (**K**) Refractive index according to wavelength of solution-processed films with various concentrations of polymer sensitizer and emitter.

The TADF polymer, poly(10-(3-(4-(8-phenyloctyl)phenyl)-5,9-dioxa-13b-boranaphtho[3,2,1-de]anthracen-7-yl)-10H-spiro[acridine-9,9′-fluorene]) (PDBA-SAF-P8P), is designed to consist of an alternating copolymer comprising a spiroacridine donor and an oxygen-bridged boron acceptor as the TADF monomer, and 4-(8-phenyloctyl)phenyl as the conjugation-breaking spacer (Fig. 1A and figs. S1 to S11).The TADF monomer has a highly twisted and rigid structure, which is expected to reduce structural disorder, thereby reducing vibrational transitions, and low energy difference between S_1_ and triplet energy level (T_1_), leading to improved luminescence efficiency. Furthermore, the incorporation of the 4-(8-phenyloctyl)phenyl unit interrupts π-conjugation along the backbone, which helps localize excited states and supress aggregation, thereby narrowing the emission bandwidth, enhancing color purity and improving film-forming ability.

The thermal stability of PDBA-SAF-P8P was confirmed by thermogravimetric analysis and differential scanning calorimetry. The polymer has a 10% weight-loss temperature > 400°C and a glass-transition temperature *T*_g_ ~ 150°C (fig. S12). These results indicate excellent thermal robustness suitable for SOLED applications.

The polymer had an absorption maximum at 310 nm and a strong photoluminescence (PL) peak at 465 nm, which are consistent with its designed donor-acceptor structure (Fig. 1B and fig. S13). The TADF unit incorporated into the synthesized polymer consists of a spiroacridine fluorene donor and an oxygen-bridged boron acceptor (Fig. 1A). The deep highest occupied molecular orbital (HOMO) level of the donor and the shallow lowest unoccupied molecular orbital (LUMO) level of the acceptor collectively contribute to realization of blue emission (*12*, *30*). Electrochemical characterization by cyclic voltammetry showed a HOMO energy level of 5.53 eV, and the LUMO energy level was estimated to be 2.46 eV (fig. S14 and table S1). These energy levels ensure appropriate alignment with adjacent charge-transport layers in simple-structured SOLED architecture. The PDBA-SAF-P8P exhibited clear TADF behavior, as evidenced by the biexponential decay profile, which includes a prompt lifetime ($\tau_{p}$ = 31.4 ns) and a delayed lifetime ($\tau_{d}$ = 1.21 μs) in time-resolved PL (TRPL) analysis (Fig. 1C and table S2).

To complete the energy transfer cascade, we paired the polymer sensitizer with a planar MR-TADF emitter. While MR-TADF emitters enable narrowband blue emission, their rigid π-conjugated structures often lead to morphological challenges under solution-processing conditions (*21*), which easily leads to poor solubility and severe aggregation during solution processing (Fig. 1D). These processes can cause inhomogeneous dispersion within the host and severe phase segregation, which can cause morphological instability and luminescence loss in solution-processed thin film devices. As a result, SOLEDs using MR-TADF emitter are particularly vulnerable to aggregation-induced luminescence quenching and self-reinforcing aggregation-induced degradation unless morphological control strategies are used (*20*–*22*).

Atomic force microscopy (AFM) analysis suggests that aggregation of *N*7,*N*7,*N*13,*N*13,5,9,11,15-octaphenyl-5,9,11,15-tetrahydro-5,9,11,15-tetraaza-19b,20b-diboradinaphtho[3,2,1-de: 1′,2′,3′ -jk]pentacene-7,13-diamine, (ν-DABNA) is less apparent at low doping levels due to its subtle expression, but that increasing the concentration revealed pronounced surface roughness, which indicates substantial aggregation and phase separation. At 10 wt % ν-DABNA loading in host matrix, the root mean square roughness *r*_RMS_ increased markedly from 0.751 to 5.754 nm; this change suggests the occurrence of dopant-induced phase separation and interfacial inhomogeneity, which degrade charge balance and radiative recombination (Fig. 1, E and F) (*20*).

In contrast, PDBA-SAF-P8P has high molecular weight and good solubility, which enables smooth film formation during solution processing. Its flexible side chains are designed to minimize aggregation-induced exciton annihilation and to promote uniform dopant dispersion within the host matrix. Incorporating this polymer sensitizer in the small-molecule EML helps suppress dopant aggregation and phase separation by diluting the dopant environment and disrupting intermolecular packing (Fig. 1, A and G). Furthermore, chain entanglement and the high *T*_g_ of the polymer contribute to increased morphological and interfacial stability. As a result, even at a high ν-DABNA doping level (10 wt %), the HF film maintains a smooth surface morphology (*r*_RMS_: 0.781 nm), which indicates effective suppression of molecular aggregation (Fig. 1, H and I).

Increasing the ν-DABNA concentration in the small-molecule host leads to progressive decrease in PL lifetimes; this trend suggests that increased dopant loadings increase exciton quenching that is induced by aggregation and Dexter-type energy transfer (DET) between adjacent emitter molecules (Fig. 1J). Increase in ν-DABNA concentration from 3 to 10 wt % also noticeably increased the refractive index *n*; this result is evidence of increased molecular packing and aggregation of the MR-TADF (Fig. 1K). In contrast, when 33 wt % of PDBA-SAF-P8P relative to the host matrix was incorporated, *n* remained nearly unchanged even at a high ν-DABNA concentrations. These results demonstrate that the polymer sensitizer not only facilitates triplet exciton harvesting and efficient energy transfer but also plays a critical morphological role, which is essential for SOLEDs (*20*, *31*, *32*). In our system, incorporation of the polymeric TADF sensitizer PDBA-SAF-P8P effectively suppresses dopant aggregation and stabilizes film morphology, as evidenced by refractive index, Raman spectroscopy, ultraviolet-visible (UV-vis) absorption, and TRPL analyses (see figs. S15 to S18).

Although the ν-DABNA concentration used in actual devices is limited to 2 to 3 wt %, aggregation-induced exciton quenching can still occur below the detection limit of conventional morphological probes. To visualize and analyze these effects, we used higher dopant concentrations as stress-test conditions, revealing that polymer incorporation effectively suppresses aggregation-related exciton loss across the entire concentration range (fig. S18).

### Self-organized polymeric HILs

In blue OLEDs, blue-emitting dopant molecules are typically dispersed in host materials that have large energy bandgap *E*_g_, to achieve efficient energy transfer to the dopants (*31*). Furthermore, due to the difficulty in forming multilayer structures in SOLEDs, a substantial energy barrier exists between the HIL and the EML. This barrier severely impedes efficient hole injection from the anode to the EML (*6*). Therefore, the HIL material of blue SOLEDs must be designed judiciously so that its electronic properties yield superior charge balance, luminous efficiency, and stability.

The commonly used polymeric HIL, poly(3,4-ethylenedioxythiophene) polystyrene sulfonate (PEDOT:PSS), has a relatively low ionization potential (IP; 5.0 ≤ IP ≤ 5.2 eV), which is markedly shallower than the HOMO levels of typical hosts for blue emitters (~6.0 eV), so the energy barrier is large at the HIL/EML interface (*33*, *34*). In addition, PEDOT:PSS is acidic, so it easily etches the underlying indium tin oxide (ITO); this process leads to diffusion of metallic species into the overlying layers, which induces severe interfacial exciton quenching (*34*). This surface quenching at the interface becomes particularly critical in devices that incorporate TADF because TADF materials have longer exciton lifetimes (≥1 μs) than fluorescence counterpart; this increases the probability of nonradiative recombination and energy loss at the HIL/EML interface (*35*, *36*).

**Fig. 2.**
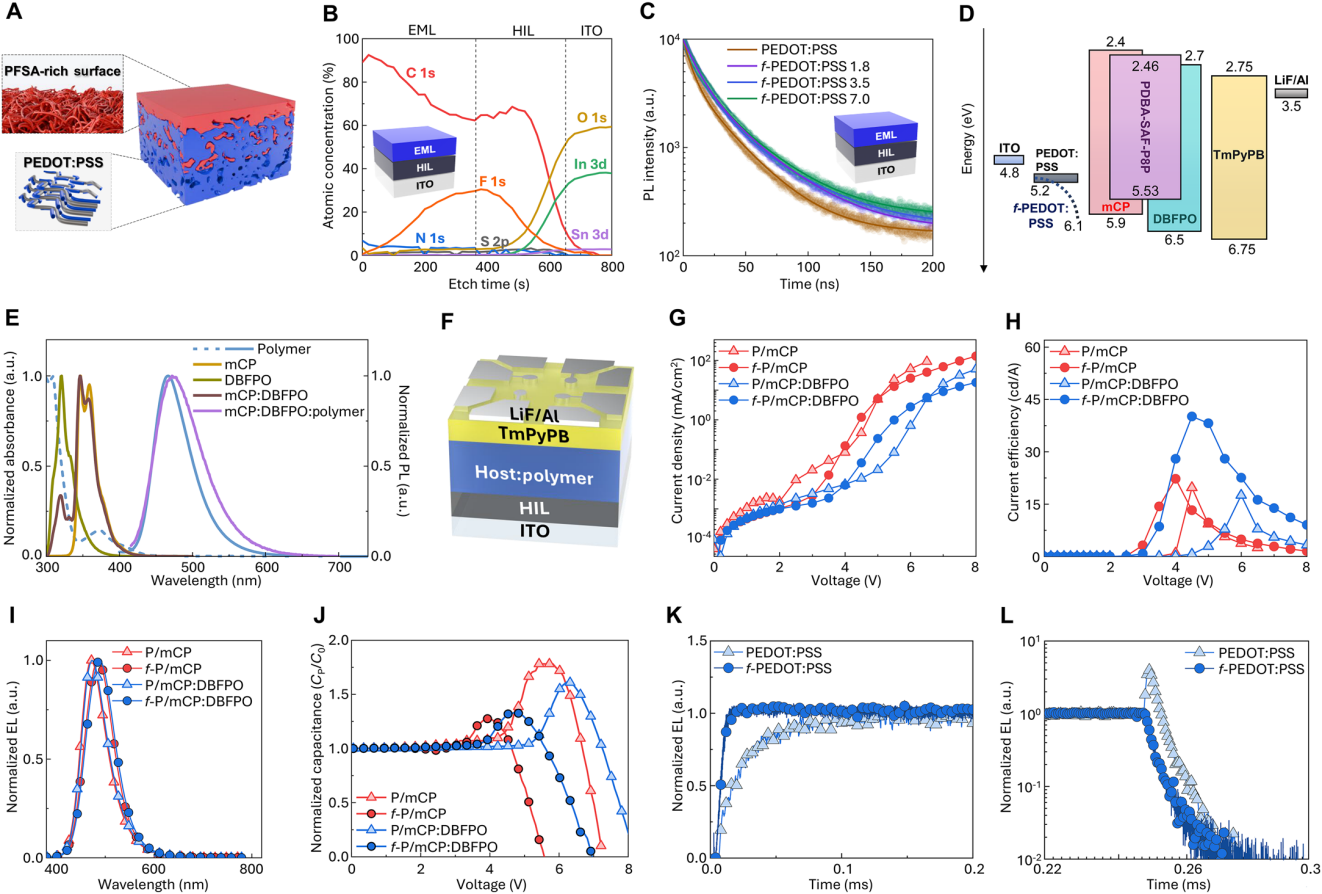
Interfacial engineering and device characteristics of polymer-based TADF OLEDs. (**A**) Schematic illustration of *f*-PEDOT:PSS structure. (**B**) Depth profile of x-ray photoelectron spectroscopy (XPS) measurements of atomic concentrations as a function of etch time for ITO/*f*-PEDOT:PSS/EML. (**C**) TRPL spectra of ITO/HIL/EML films using various HILs; the number following “*f*-PEDOT:PSS” represents the weight ratio of PFSA to PEDOT. (**D**) Energy level diagram of SOLEDs incorporating 1,3-di(9H-carbazol-9-yl)benzene (mCP), 2,8-bis(diphenylphosphoryl)dibenzo[b,d]furan (DBFPO), and PDBA-SAF-P8P in the EML. (**E**) Normalized UV-vis absorption and PL spectra of solution-processed films containing mCP, DBFPO, PDBA-SAF-P8P, and their blends. (**F**) Device structure of SOLEDs using PDBA-SAF-P8P as emitter. (**G**) Current density versus voltage, (**H**) current efficiency (CE) versus voltage, (**I**) normalized electroluminescence (EL) spectra, and (**J**) normalized capacitance versus voltage characteristics of SOLEDs using mCP single host and mCP:DBFPO mixed host with PEDOT:PSS and *f*-PEDOT:PSS. Transient EL (trEL) (**K**) rising and (**L**) decaying characteristics of mCP:DBFPO mixed-host SOLEDs with PEDOT:PSS and *f*-PEDOT:PSS.

To overcome these challenges, we used a self-organized, surface-fluorinated polymeric HIL (*f*-PEDOT:PSS), composed of PEDOT:PSS and a perfluorinated sulfonic acid (PFSA) that has a lower surface energy and higher IP than PEDOT:PSS (*20*, *34*). These properties enable spontaneous self-organization of these two polymer materials during film formation. Because of its lower surface energy (~20 mN/m) compared to PEDOT:PSS (~65 mN/m) (*37*), PFSA preferentially migrates to the film surface, resulting in vertical compositional stratification and a gradient in work function that increases from the anode toward the EML (Fig. 2A). In addition, the surface-enriched PFSA layers effectively block diffusion of metal atoms to the proximity of the EML (*20*). This approach minimizes nonradiative recombination at the HIL/EML interface, creates a graded energy landscape to facilitate hole injection, and forms a protective interfacial barrier against surface exciton quenching and interlayer mixing during solution processing (*20*, *31*, *34*).

X-ray photoelectron spectroscopy (XPS) depth profiling for ITO/HIL/EML stack exhibited a clear fluorine gradient and no detectable indium above the PFSA-rich region; these observations confirm surface enrichment of PFSA and efficient blocking of metal diffusion (Fig. 2B and fig. S19).

As the amount of PFSA in the HIL increased, the surface-enriched insulating chains become thicker and keep excitons away from the PEDOT:PSS or diffused metal species; these phenomena increase average PL lifetime $\tau_{\text{avg}}$ (Fig. 2C). $\tau_{\text{avg}}$ increased from 18.13 ns on the PEDOT:PSS to 23.65 ns on the *f*-PEDOT:PSS 7.0 (PFSA:PDOT = 7:1 w/w); this trend suggests a gradual reduction in exciton quenching at the interface.

The work function and the HOMO energy level gradually increased toward the surface of the HIL film, to 6.1 eV in the topmost surface of *f*-PEDOT:PSS, as confirmed by ultraviolet photoelectron spectroscopy (UPS) depth profiling (fig. S20). This energy-level gradient facilitates hole injection into the EML by Fowler-Nordheim tunneling and thus effectively lowers the energy barrier at the HIL/EML interface in the simple device architecture (*38*).

### Blue polymer OLEDs that use PDBA-SAF-P8P

To evaluate the electroluminescence (EL) properties of the PDBA-SAF-P8P as a TADF emitter, we fabricated solution-processed blue-emitting TADF OLEDs that incorporate a hybrid EML (Fig. 2D). In the architecture ITO/HIL (40 nm)/EML (30 nm)/1,3,5-tri(m-pyridin-3-ylphenyl)benzene (TmPyPB) (50 nm)/LiF (1 nm)/Al (100 nm), PEDOT:PSS or *f*-PEDOT:PSS was used as HIL. In the EML, small molecular organic semiconductor materials were used as a host for the TADF polymer guest (Fig. 2, D to F).

To quantify the synergistic effects of host polarity and polymeric HIL properties on TADF OLED characteristics, we first compared devices that included different combinations of host materials and HILs. The single-host EML composed of the hole-transporting host 4,4′,4″-tri-9-carbazolyltriphenylamine (TCTA) or the bipolar-transporting host 1,3-di(9H-carbazol-9-yl)benzene (mCP) was investigated (figs. S21 to S24). When TCTA was host material, its relatively shallow HOMO energy level (~5.7 eV) resulted in a relatively low hole-injection energy barrier height $\phi_{h}$. However, TCTA has a large imbalance between hole mobility ($\mu_{h}=3\times 10^{-3}{\text{cm}}^{2}V^{-1}s^{-1}$) and electron mobility ($\mu_{e}<10^{-8}{\text{cm}}^{2}V^{-1}s^{-1}$) (*39*, *40*); this difference leads to poor charge balance and consequently to low luminous efficiency (figs. S21 to S24). In contrast, mCP has a deeper HOMO energy level (~5.9 eV) and consequently a larger $\phi_{h}$ than TCTA, but relatively ambipolar transport characteristics ($\mu_{h}=1.2\times 10^{-4}{\text{cm}}^{2}V^{-1}s^{-1}$ and $\mu_{e}=5.1\times 10^{-6}{\text{cm}}^{2}V^{-1}s^{-1}$) (*41*) increase charge balance and thereby increase device efficiency (figs. S21 to S24). Devices that used TCTA had low threshold voltage *V*_th_ due to their shallow HOMO, whereas devices that used mCP suffered from high *V*_th_ when paired with conventional PEDOT:PSS HIL. This high *V*_th_ was substantially reduced using the *f*-PEDOT:PSS, which improved hole injection into the deep-HOMO host mCP (Fig. 2G). In addition, mCP has a higher T_1_ = ~3.13 eV than TCTA (T_1_ ~ 2.70 eV) (*42*, *43*) and therefore effectively blocks backward energy transfer from the emitter, thereby increasing the efficiency of mCP host–based devices.

To improve charge balance, we used a mixed-host system composed of 85 wt % hole-dominant ambipolar host mCP and 15 wt % electron-transporting host 2,8-bis(diphenylphosphoryl)dibenzo[b,d]furan (DBFPO) (HOMO level ~ 6.5 eV) (*44*); this combination exploits the hole-transporting properties of mCP and the electron-transporting properties of DBFPO. However, in this configuration, the host materials have a much deeper HOMO level ($\phi_{h}$ > 1.3 eV) than mCP- or TCTA-only device and therefore severely hinder hole injection from PEDOT:PSS. As a result, devices with PEDOT:PSS had higher *V*_th_ (~4.5 V) and relatively poor luminous efficiency (~17.5 cd A^−1^) than those with *f*-PEDOT:PSS, which had a deeper surface IP (~6.1 eV) and lower $\phi_{h}$ (~0.4 eV) and therefore achieved efficient hole injection (*V*_th_: ~3.5 V) and a substantially higher luminous efficiency of 40.1 cd A^−1^ (Fig. 2, D and F to I). These results can also be attributed to the reduced hole transport capacity of the mixed host upon introduction of DBFPO, which increases the sensitivity of the device to increases in hole injection. For comparison, mCP was also mixed with bis[2-(diphenylphosphino)phenyl]ether oxide (DPEPO), an electron-transporting host, but its lower electron-transporting ability ($\mu_{e}=5.6\times 10^{-6}{\text{cm}}^{2}V^{-1}s^{-1}$) (*45*) compared to DBFPO led to insufficient charge balance and limited improvement in luminous efficiency (figs. S25 to S27).

To verify the improved hole injection and recombination dynamics, we analyzed capacitance-voltage (*C*-*V*) characteristics (Fig. 2J). *V*_th_ in *C*-*V* characterization was defined as the voltage at which capacitance begins to increase due to majority carrier injection. In devices that use mCP or mCP:DBFPO, *V*_th_ was obviously reduced when *f*-PEDOT:PSS was applied; this result indicates increased efficiency of hole injection at a reduced applied bias (Fig. 2J). Notably, the reduction in *V*_th_ was more pronounced in mixed-host devices than in single-host devices; this difference is consistent with the *V*_th_ trend in *J*-*V* curves. Furthermore, in both host systems, the use of *f*-PEDOT:PSS led to a decrease in both the peak capacitance *C*_peak_ and its corresponding voltage *V*_peak_ and resulted in a steeper capacitance drop at >*V*_peak_. These results suggest improvements in the balance of charge transport and of radiative recombination dynamics within SOLEDs (Fig. 2J).

EL rise *t*_r_, defined as the time taken for EL emission to reach 80% of its maximum intensity in transient EL (trEL) characterization, was notably shorter (~10 μs) in the device that used *f*-PEDOT:PSS than in the device that used PEDOT:PSS (~39 μs). This difference suggests that the *f*-PEDOT:PSS improved hole injection and reduced the electron-transit distance to the recombination zone (Fig. 2K) (*46*).

Devices that incorporated PEDOT:PSS showed a trEL overshoot immediately following termination of the square voltage pulse, whereas devices that incorporated *f*-PEDOT:PSS did not show this phenomenon (Fig. 2L). The overshoot may be a result of hole trapping at the PEDOT:PSS/EML interface, as a consequence of the substantial energy barrier at the interface. When the voltage is switched off, these trapped charges escape and recombine with counter charge carriers, and this generates delayed EL spikes (*47*). These results indicate that the complementary charge transport properties of mCP and DBFPO contribute to improved charge balance in the mixed-host system, in which the hole injection barrier imposed by the deep HOMO of DBFPO is effectively alleviated by the *f*-PEDOT:PSS use.

### Polymer-TADF–sensitized fluorescence

To achieve high color purity and full exciton utilization in SOLEDs, the TADF-sensitized fluorescence strategy is a promising approach that synergistically combines TADF sensitizers with narrowband MR-TADF emitters. In this architecture, RISC in the TADF sensitizer upconverts electrically generated triplet excitons to S_1_, from which they are transferred to the terminal emitter. This process enables 100% exciton harvesting while preserving the sharp emission profiles and color purity of MR-TADF emitters (*10*, *14*, *15*). Notably, rapid FRET to the terminal emitter narrows the TADF sensitizer’s broad emission and increases its relatively low radiative recombination rate. As a result, the narrowband emission of the terminal emitter is preserved, so the HF system achieves efficient energy transfer, fast radiative decay, and a narrow EL bandwidth.

**Fig. 3.**
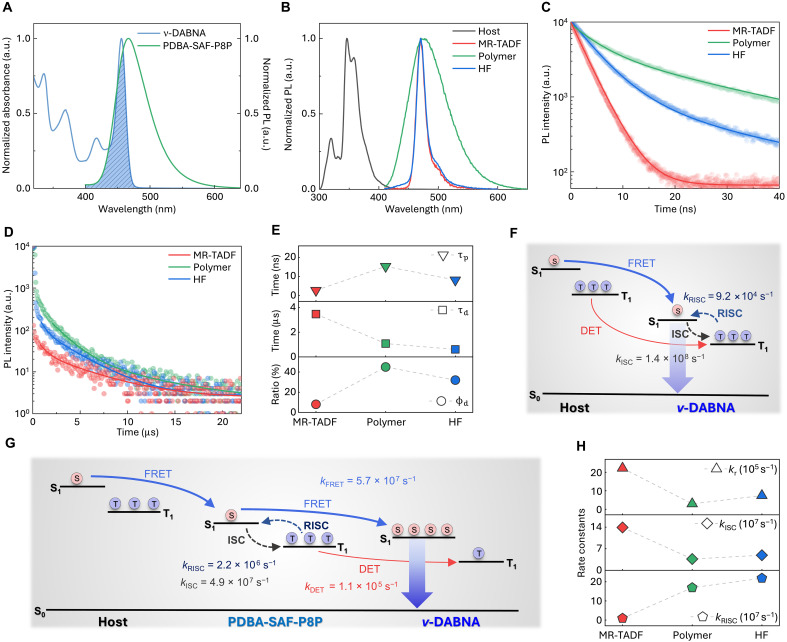
Photophysical properties and energy transfer dynamics of polymer-sensitized hyperfluorescence. (**A**) Normalized UV-vis absorption of ν-DABNA and PL spectra of PDBA-SAF-P8P in toluene. (**B**) Normalized PL spectra of solution-processed films of MR-TADF [mCP:DBFPO (15 wt %):ν-DABNA (2 wt %)], TADF polymer [mCP:DBFPO (15 wt %):PDBA-SAF-P8P (33 wt %)], and HF [mCP:DBFPO (15 wt %):PDBA-SAF-P8P (33 wt %):ν-DABNA (2 wt %)]. TRPL spectra of (**C**) prompt fluorescence (PF) and (**D**) delayed fluorescence (DF) of solution-processed films. (**E**) PF and DF lifetimes and the DF contribution ratios of solution-processed films. Schematic illustrations of energy transfer process in (**F**) host: MR-TADF emitter system and (**G**) polymer-sensitized HF emission system with rate constants derived from the emission decays. In the HF system, dominant exciton dynamics are governed by the polymer sensitizer, while possible RISC processes within MR-TADF are considered negligible under these conditions. (**H**) Rate constants of radiative decay, intersystem crossing (ISC), andRISC of solution-processed MR-TADF, TADF polymer, and HF films.

Here, we used ν-DABNA as the terminal emitter in conjunction with a polymeric TADF sensitization. The PL spectrum of the polymeric TADF sensitizer and the absorption profile of ν-DABNA have a substantial spectral overlap, which indicates that efficient FRET from polymer to ν-DABNA is available (Fig. 3A). When doped into mCP:DBFPO mixed-host matrix, PDBA-SAF-P8P had an emission peak at 477 nm [full width at half maximum (FWHM): 77 nm], and ν-DABNA had an emission peak at 470 nm (FWHM: 19 nm). The HF film [mCP:DBFPO (15 wt %):PDBA-SAF-P8P (33 wt %):ν-DABNA (2 wt %)] had a sharp emission peak at 470 nm (FWHM: 19 nm), which was identical to the emission of the MR-TADF film [mCP:DBFPO (15 wt %):ν-DABNA (2 wt %)]. This result confirms that efficient FRET from the polymer sensitizer enables emission from the terminal MR-TADF emitter (Fig. 3B and fig. S28).

To further investigate the energy transfer and emission dynamics in the hybrid HF EML, we conducted TRPL measurement for doped films with or without polymer or ν-DABNA (Fig. 3, C to E, and Table 1). TRPL measurements revealed distinct exciton dynamics in the three different EML systems. The TADF polymer film had a long prompt lifetime $\tau_{p}$ (15.29 ns) and short delayed lifetime $\tau_{d}$ (1.08 μs), along with a high delayed component fraction $\phi_{d}$ (0.45), which is characteristics of efficient TADF behavior. In contrast, the MR-TADF film showed minimal delayed emission, consistent with relatively poor triplet-harvesting characteristics ($\tau_{p}$: 2.77 ns, $\tau_{d}$: 3.47 μs, and $\phi_{d}$: 0.08). Notably, the HF film demonstrated reduced $\tau_{p}$ (8.17 ns) and intermediate $\phi_{d}$ (0.32); these traits indicate successful triplet harvesting by the polymer sensitizer, followed by efficient energy transfer to the MR-TADF emitter. The delayed emission decay profile of the HF film resembles that of the pure polymer sensitizer film; this similarity suggests that the triplet excitons generated in the polymer undergo fast RISC and are subsequently transferred to the S_1_ of ν-DABNA (Fig. 3, C to E) (*10*).

**Table 1.** Photophysical properties and rate constants from the emission decays of solution-processed films.

Film	$\phi_{\text{PL}}$ *	$\phi_{p}$ ^†^	$\phi_{d}$ ^†^	$\tau_{p}$^‡^ [ns]	$\tau_{d}$^‡^ [μs]	*k*_r_^§^ [10^7^ s^−1^]	*k*_ISC_^‖^ [10^7^ s^−1^]	*k*_RISC_^¶^ [10^5^ s^−1^]	*k*_FRET_^#^ [10^7^ s^−1^]	*k*_DET_^#^ [10^5^ s^−1^]
MR-TADF	0.69	0.61	0.08	2.77	3.47	22.2	13.9	0.92	–	–
TADF polymer	0.89	0.44	0.45	15.29	1.08	2.92	3.66	16.91	–	–
HF	0.92	0.60	0.32	8.17	0.61	7.39	4.89	21.86	5.70	1.07

* Photoluminescence quantum yield (PLQY) in film, excited at 310 nm.

† Combined contribution of prompt fluorescence $\phi_{p}$ component and delayed fluorescence $\phi_{d}$ component to the PLQY for the thin film.

‡ Prompt fluorescence $\tau_{p}$ and delayed fluorescence $\tau_{d}$ lifetime.

§ Rate constants of radiative decay.

‖ Rate constants of ISC.

¶ Rate constants of RISC.

# Rate constants of FRET and DET.

The kinetic analysis revealed that the TADF polymer film had high photoluminescence quantum yield (PLQY; 0.89) and fast RISC rate *k*_RISC_ of 1.69 × 10^6^ s^−1^; these traits are characteristic of efficient TADF behavior. In contrast, the MR-TADF film showed relatively low PLQY (0.69) and slow *k*_RISC_ (9.2 × 10^4^ s^−1^), which are consistent with relatively poor triplet utilization compared to that of TADF polymer (Fig. 3F and Table 1). Notably, the HF film had a high FRET rate *k*_FRET_ (5.70 × 10^7^ s^−1^) and low DET rate *k*_DET_ (1.07 × 10^5^ s^−1^); these results confirm that the polymer sensitizer efficiently transfers upconverted excitons to the MR-TADF emitter by singlet-mediated efficient FRET (Fig. 3, G and H, and Table 1). This energy transfer pathway minimizes unwanted Dexter-type triplet-triplet interactions at the MR-TADF sites and thereby suppresses quenching and exciton-exciton annihilation that are induced by triplet accumulation while effectively promoting S_1_ emission from the MR-TADF and increasing overall PLQY to 0.92 in HF EML (Fig. 3, F to H). Given the high radiative decay rate *k*_r_ of ν-DABNA (2.22 × 10^8^ s^−1^), the transferred singlet excitons are rapidly emitted as fluorescence. Notably, the *k*_FRET_ estimated from $\tau_{p}$ may underestimate the actual exciton transfer because fast *k*_RISC_ and $\phi_{d}$ enable increased cumulative energy transfer by the delayed component (*11*). These findings collectively demonstrate the strategic benefit of combining a high-*k*_RISC_ polymer sensitizer with a high-*k*_r_ MR-TADF emitter to enable both efficient exciton harvesting and radiative emission while suppressing energy losses by DET and thereby fulfilling the key requirements for high-efficiency HF OLEDs.

### Blue HF SOLEDs with polymer-sensitized hybrid EML

**Fig. 4.**
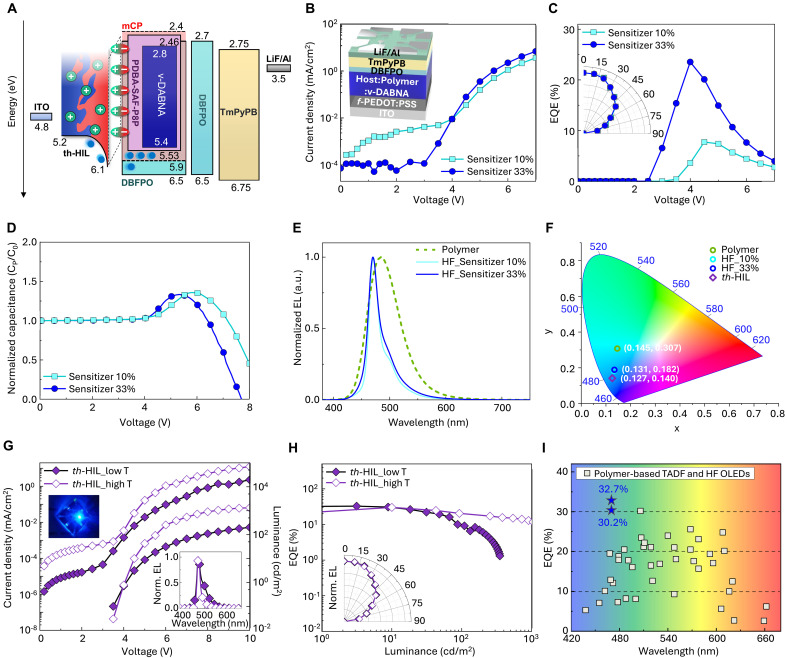
Device performance of polymer-sensitized HF SOLEDs. (**A**) Energy level diagram of SOLEDs incorporating a hybrid HF EML, with optimized architectures including ternary hybrid HIL (*th*-HIL) and DBFPO buffer layer. (**B**) Current density versus voltage (inset: device structure), (**C**) external quantum efficiency (EQE) versus voltage (inset: normalized angle-dependent EL intensity), and (**D**) normalized capacitance versus voltage characteristics of SOLEDs using HF EML with sensitizer concentrations of 10 and 33 wt %. (**E**) Normalized EL spectra of SOLEDs with different EML compositions: polymer [mCP:DBFPO (15 wt %):PDBA-SAF-P8P (33 wt %)] and HF [mCP:DBFPO (15 wt %):PDBA-SAF-P8P:ν-DABNA (2 wt %)] containing sensitizer concentrations of 10 and 33 wt %. (**F**) International Commission on Illumination (CIE) coordinates of SOLEDs with different EML compositions and SOLEDs using *th*-HIL. (**G**) Current density and luminance versus voltage characteristics of SOLEDs using *th*-HIL (inset: optical image of operating device and normalized EL spectra of SOLEDs using *th*-HIL). (**H**) EQE versus luminance characteristics of SOLEDs using *th*-HIL (inset: normalized angle-dependent EL intensity). (**I**) Comparison of reported EQE of polymer-based TADF or HF OLEDs across the entire visible spectrum from 2016 to present, where filled star (★) denotes this work and open squares (□) denote literature values.

To exploit the excellent photophysical properties of the hybrid HF system, we fabricated blue HF SOLEDs to evaluate their EL characteristics using the optimized device architecture (figs. S29 to S31). A thin DBFPO interlayer was introduced as an exciton buffer to prevent undesired exciton annihilation or field-assisted interface quenching at the EML/electron transport layer (ETL) and thus to achieve increased luminous efficiency (Fig. 4A and fig. S32).

In the *J*-*V* characteristics, the HF SOLED device with 33 wt % polymeric TADF sensitizer (relative to small molecular host matrix) had higher *J* at a given applied bias than did the 10 wt % counterpart; this comparison indicates that the polymer sensitizer contributes to facilitated charge-transport properties in the hybrid HF EML (Fig. 4B). The device with 33 wt % sensitizer achieved a higher current efficiency (CE) of 30.8 cd A^−1^ and external quantum efficiency (EQE) of 23.5% than those of the device with 10 wt % (9.4 cd A^−1^ CE and 7.7% EQE) and than those of the TADF OLEDs that used the polymer sensitizer as an emitter (18.6% EQE) (Fig. 4C and fig. S33).

Because of the deep HOMO energy level (~6.5 eV) of the optimized mCP:DBFPO mixed host, hole injection from the HIL into the hybrid HF EML requires tunneling across a substantial $\phi_{h}$ and a high operating voltage. However, when a substantial fraction of the polymer sensitizer that has moderate HOMO energy level (5.53 eV) is incorporated into the EML, hole carriers can be directly injected into the polymer HOMO levels from the conductive part of the HIL. This process facilitates hole injection across the interface and transport through polymer chains in EML and effectively mitigates the injection barrier and improves charge balance within the SOLED.

*C*-*V* analysis also revealed that devices with 33 wt % sensitizer had a lower *V*_th_ and steeper capacitance drop after *V*_peak_ compared to those with 10 wt % sensitizer; these changes indicate improved charge injection and increased efficiency of recombination dynamics (Fig. 4D). In addition, the 33 wt % sensitizer content provides more triplet exciton harvesting and RISC than the 10 wt % content and thereby further promotes singlet exciton formation in MR-TADF by efficient FRET. These results highlight the polymer’s dual optoelectronic effects of supporting improvement in the balance of charge transport and in exciton management in the HF SOLEDs.

The EL spectra of the devices also showed clear spectral evolution when ν-DABNA was used as a terminal emitter. In the TADF polymer device, the peak emission wavelength was 484 nm, and in the HF devices, the peak shifted to 470 nm, which closely matches the spectral characteristics of ν-DABNA (Fig. 4E). FWHM also substantially narrowed from 67 to 25 nm upon introduction of the HF architecture; this change indicates successful spectral refinement typically associated with MR-TADF emission. The International Commission on Illumination (CIE) coordinates also shifted from (0.145, 0.307) for the polymer-only device to (0.131, 0.182) for the HF devices (Fig. 4F). Despite the higher sensitizer loading in the 33 wt % device, no substantial spectral broadening was observed compared to the 10 wt % device. This can be attributed to the uniform dispersion and strong aggregation resistance of the polymer-sensitized HF EML film, along with the efficient energy transfer design of the hybrid HF system.

To further increase device efficiency, we also used a molecular-engineering strategy to induce self-organization in the polymeric HIL by incorporating an organic/inorganic ternary hybrid system. Although *f*-PEDOT:PSS improves interfacial stability through PFSA-enriched surface formation (*20*, *46*, *48*), excessive PFSA segregation can introduce an insulating surface layer that limits efficient hole injection (figs. S34 and S35). To overcome the injection barrier associated with insulating PFSA surface layers in *f*-PEDOT:PSS, we developed a ternary hybrid HIL (*th*-HIL) incorporating Ni^2+^, which forms Ni-sulfonate complexes during film formation. This hybridization induces finer surface morphology and coherent interfacial dipole alignment, leading to improved energetic alignment and improved hole injection (Fig. 4A and figs. S36 and S37). This *th*-HIL spontaneously forms a stratified architecture by self-organization during solution processing.

When implemented in the hybrid blue-emitting HF SOLEDs, the *th*-HIL led to an increase in luminous efficiency (Fig. 4, G to I). CE increased from 30.8 to 49.4 cd A^−1^, and EQE increased from 23.5 to 32.7% (Fig. 4, G and H, and fig. S38). The EQEs were calculated using angular EL profile of each SOLED (Fig. 4H, inset). This pronounced improvement is attributed to the synergistic effects of increased hole injection, increased optical outcoupling, and reduced optical losses enabled by the low *n* of *th*-HIL (fig. S39) (*31*). To address the trade-off between peak luminous efficiency and luminance, we systematically optimized the hybrid EML structure by adjusting the postannealing temperature and HIL composition. As a result, the optimized devices with elevated annealing temperature exhibited higher luminance (~1700 cd m^−2^), suppressed efficiency roll-off, and improved color purity with a reduced FWHM of 18 nm (CIE coordinates: 0.127, 0.140) while maintaining a high peak EQE of 30.2% (Fig. 4, F to H, and figs. S40 and S41). These improvements originate from enhanced molecular packing and interfacial alignment within the hybrid EML, promoting more effective exciton recombination on the MR-TADF emitter. To the best of our knowledge, this work presents the first demonstration of a blue-emitting HF OLED enabled by polymer sensitization and also the highest EQE reported for polymer-based TADF or HF OLEDs across the entire visible spectrum (Fig. 4I, fig. S42, and tables S3 and S4).

While the polymer-sensitized devices demonstrated high efficiency and improved morphological stability, their maximum luminance value remains somewhat lower than that of state-of-the-art all–small-molecule SOLEDs (*49*, *50*). In addition, although improvement in operational stability was observed compared to the all–small-molecule control, the absolute lifetime values are still limited (fig. S41). These observations highlight that while the hybrid polymeric architecture effectively mitigates aggregation and interfacial degradation, the operational stability remains fundamentally limited by the intrinsic excited-state reactivity and degradation susceptibility of deep-blue MR-TADF emitters such as ν-DABNA (*21*, *22*). Nevertheless, our results demonstrate that rational design strategies including polymer backbone engineering, interfacial tuning, and optimized thermal treatment can mitigate these limitations and enable further improvements in both luminance and device lifetime. Future integration of stable terminal emitters and tailored encapsulation schemes may further extend the operational stability of this platform. Although several all–small-molecule solution-processed blue OLEDs have recently achieved excellent efficiencies (fig. S43 and table S5) (*22*, *49*, *51*), polymer-based systems offer a fundamentally different design space. Rather than replacing small-molecule systems, our approach complements them by addressing specific morphological and mechanical limitations associated with planar small-molecule emitters in solution-processed environments while enabling new directions in material and device design. In this work, we demonstrate the first polymer-sensitized blue HF OLED, achieving near state-of-the-art efficiency together with ultrahigh color purity. Beyond efficiency, the polymer-hybrid architecture provides additional advantages in morphology control, processing stability, and mechanical compliance, highlighting its potential as a complementary and scalable platform for next-generation SOLEDs (fig. S44).

## DISCUSSION

We developed high-efficiency solution-processed blue-emitting HF OLEDs using a polymeric TADF sensitizer PDBA-SAF-P8P and a narrowband MR-TADF terminal emitter in a hybrid EML. The polymer sensitizer, designed with controlled conjugation and high solubility, achieved efficient triplet harvesting, fast RISC, and excellent morphological compatibility and thereby enabled strong FRET to the terminal emitter while preventing aggregation and phase separation even at high doping concentrations.

Incorporation of the polymer sensitizer into the small-molecule host matrix forms a functionally asymmetric hybrid EML, in which the polymer network improves film-forming properties, suppresses crystallization, and provides a favorable energetic and spatial environment for diffusion and transfer of excitons. This hybrid architecture thus combines the processing and morphological stability of polymers with the spectral purity and tunability of molecular emitters, and this improves exciton management and increases device performance.

We further increased charge injection and exciton confinement by introducing a self-organized surface-fluorinated HIL and a hybrid HIL ; these additions facilitated effective energy level alignment and suppressed exciton quenching at the interface. The resulting devices achieved a maximum EQE of 32.7% with a narrow emission bandwidth (FWHM: 25 nm),and further optimization yielded improved color purity with a FWHM of 18 nm at an EQE of 30.2%.

Our findings establish a generalizable multifunctional hybrid strategy to design highly efficient HF in SOLEDs, in which the polymeric TADF sensitizer serves both as a triplet harvester and as a morphological stabilizer and charge transport mediator, while the self-organized HILs simultaneously ensure efficient hole injection, energy level alignment, and interfacial passivation. This work demonstrates the synergistic advantages of combining a polymer/small-molecule hybrid HF EML architecture with interfacially engineered HILs and offering a generalizable strategy for realizing high-efficiency blue-emitting HF OLEDs by scalable solution processing.

## MATERIALS AND METHODS

### Materials

BBr_3_ was purchased from Sigma-Aldrich, and common reagents were purchased from Sigma-Aldrich, Tokyo Chemical Industry, or Alfa Aesar and were used without additional purification. Anhydrous toluene was purchased from Sigma-Aldrich, and common organic solvents were purchased from Daejung Chemical & Metal Co. Ltd. 1,8-bis(4-(4,4,5,5-tetramethyl-1,3,2-dioxaborolan-2-yl)phenyl)octane was synthesized using procedures previously (*52*).

### Device fabrication

Glass substrate patterned with ITO was sequentially sonicated in acetone and isopropyl alcohol for 15 min each and then treated with UV ozone for 20 min. Polymeric HIL was then spin coated on the glass/ITO substrates. The *f*-PEDOT:PSS was prepared by mixing PEDOT:PSS (CLEVIOS P VP AI 4083; PEDOT:PSS ratio = 1:6 w:w) with tetrafluoroethylene-perfluoro-3,6-dioxa-4-methyl-7-octene-sulfonic acid copolymer (Sigma-Aldrich) at various mass ratios. The substrates were then annealed at 150°C for 30 min. Substrates were transferred to a nitrogen-filled glove box, in which the EML was spin coated onto the HIL film at 4000 rpm for 60 s. The EML solution was prepared by dissolving host, PDBA-SAF-P8P and ν-DABNA molecules, in tetrahydrofuran (anhydrous, ≥99.9%; Sigma-Aldrich). mCP, DBFPO, and ν-DABNA were purchased from Nichem Fine Technology. The EML was thermally annealed at 75°C for 30 min. The devices were completed by sequential vacuum deposition of DBFPO (5 nm, ≥99.9%; Nichem Fine Technology) and TmPyPB (45 nm, >99.9%; Nichem Fine Technology) as the ETL, LiF (1 nm, ≥99.99%; Sigma-Aldrich), and Al (100 nm; iTASCO) under high vacuum (≤5.0 × 10^−7^ torr).

### Characterizations

Current-voltage-luminance (*J*-*V*-*L*) characteristics and luminous efficiencies of devices were measured using a source-measurement unit (Keithley 2450), and a spectroradiometer (Komica-Minolta CS-2000). Capacitance-voltage (*C*-*V*) characteristics were measured using an electrochemical impedance spectroscope (BioLogic SP-300), applying a voltage sweep from 0 to 10 V at a constant frequency of 1000 Hz. Steady-state PL measurements were conducted using Jasco FP-8500 fluorometer with a xenon lamp as the light source, and a PicoQuant Fluotime 300 equipped with a 405-nm laser. TRPL measurements were performed using the PicoQuant Fluotime 300 equipped with a 405-nm laser. UV-vis absorption spectra were obtained using Jasco V-730 UV-vis spectrophotometers. The PLQYs of organic films were measured using Jasco FP-8500 fluorometer with a 100-mm integrating sphere (ILF-835) and analyzed using the Jasco Spectra Manager software. Refractive indexes of films were measured using a spectroscopic ellipsometer [Elli-SE(UV)-FM8, Ellipso Technology]. trEL characteristics were measured using a pulse generator (HP 8116A; 90 ms, 10-Hz frequency), photomultiplier tube (PMTSS, Thorlabs), and oscilloscope (Agilent Infiniium 54832D MSO). Surface morphology was analyzed using an AFM (NX10, Park Systems). UPS measurements were conducted using a Theta Probe (Thermo Fisher Scientific) equipped with a He I (21.2 eV) source, with a bias of −10 eV applied to the sample. XPS (K-Alpha plus, Thermo Fisher Scientific) was conducted using a monochromatic Al Kα source (1486.6 eV). Depth profiling was conducted using Ar^+^ sputtering (1 keV, 70 s).

## Supplementary Materials

### This PDF file includes:

Supplementary Text Figs. S1 to S44 Tables S1 to S5 References
