# Supplementary material for: Polymer-Sensitized Hybrid Design Strategy for High-Efficiency Blue Hyperfluorescent OLEDs
Source: Sci Adv. 2026 Apr 10;12(15):eaee0158. doi: 10.1126/sciadv.aee0158 (PMC13068077; doi:10.1126/sciadv.aee0158)
Supplement: Supplementary file 1 — Supplementary Text Figs. S1 to S44 Tables S1 to S5 References [file sciadv.aee0158_sm.pdf]

Science Advances
AAAS

Supplementary Materials for

Polymer-Sensitized Hybrid Design Strategy for High-Efficiency Blue Hyperfluorescent OLEDs

Junwon Jeon *et al.*

Corresponding author: Yun-Hi Kim, ykim@gnu.ac.kr; Tae-Hee Han, taeheehan@hanyang.ac.kr

Sci. Adv. **12**, eaee0158 (2026)
DOI: 10.1126/sciadv.aee0158

This PDF file includes:

Supplementary Text
Figs. S1 to S44
Tables S1 to S5
References

I. Synthesis and Characterization

General methods: All reactions were strictly performed under nitrogen conditions. Proton nuclear magnetic resonance (^{1}H NMR) and carbon nuclear magnetic resonance (^{13}C NMR) spectra were measured on a Bruker DRX 400 MHz spectrometer. High-resolution mass spectra (HRMS) were measured by the quadrupole time-of-flight (QTOF) methods using a Xevo G2-XS Tof. Thermogravimetric analysis (TGA) was performed using a TA 2050 TGA thermogravimetric analyzer under a nitrogen atmosphere. The sample was heated at 10 °C/min from 50 ºC to 800 ºC. Differential scanning calorimetry (DSC) was performed using a TA Instruments 2100 DSC under a nitrogen atmosphere. The sample was heated at 10 °C/min from 0 °C to 300 °C. Absorption spectrum was measured using a PerkinElmer LAMBDA-900 UV/vis/IR spectrophotometer. The solution room temperature (300 K) and low temperature (77 K) PL spectra were measured by a LS-50B luminescence spectrophotometer. Cyclic voltammetry was measured on a ZIVE SP1, Wonatech at a room temperature in a 0.1 M solution of tetrabutylammonium perchrolate (Bu_4NClO_4) in CH_2Cl_2 at a scanning rate of 20 mV/s. A Pt wire was used as the counter electrode and an Ag/Ag^+ electrode was used as the reference electrode. The redox potential for the target material was reported regarding the ferrocene/ferrocenium (Fc/Fc+) redox couple. The number-average molecular weights (Mn) were used by GPC using the Waters Breeze Isocratic system, THF as the eluent with a flow rate of 1.0 mL/min.

The PDBA-SAF-P8P, was synthesized using Suzuki-Miyaura polycondensation between 10-(3,11-dibromo-5,9-dioxa-13b-boranaphtho[3,2,1-de]anthracen-7-yl)-10H-spiro[acridine-9,9'-fluorene] and 1,8-bis(4-(4,4,5,5-tetramethyl-1,3,2-dioxaborolan-2-yl)phenyl)octane (Fig. 1A and figs. S1 to S11).

Synthesis of 10-(4-bromo-3,5-difluorophenyl)-10H-spiro[acridine-9,9'-fluorene] (3): 2-Bromo-1,3-difluoro-5-iodobenzene (5.0 g, 15.67 mmol), 10*H*-spiro[acridine-9,9'-fluorene] (4.7 g, 14.25 mmol), sodium *tert*-butoxide (2.1 g, 21.3 mmol), CuI (0.41 g, 2.13 mmol), and trans-1,2-diaminocyclohexane (0.73 g, 6.41 mmol) were dissolved in anhydrous toluene (100.0 mL) under a nitrogen atmosphere. After stirring at 120 °C for 12 h, the reaction mixture was filtered through a pad of Florisil® using toluene as an eluent. After the solvent was evaporated, the crude product was washed with hexane. After purification, the product was obtained as a white solid (yield: 4.8 g, 65%). ^{1}H NMR (400 MHz, CD_2Cl_2) δ 7.89 (dt, J = 7.6, 0.9 Hz, 2H), 7.45 (td, J = 7.4, 1.4 Hz, 2H), 7.41 – 7.36 (m, 2H), 7.32 (td, J = 7.4, 1.3 Hz, 2H), 7.29 – 7.21 (m, 2H), 7.02 (ddd, J = 8.5, 7.1, 1.6 Hz, 2H), 6.66 (td, J = 7.4, 1.3 Hz, 2H), 6.50 – 6.39 (m, 4H). ^{13}C NMR (101 MHz, CD_2Cl_2) δ 156.33, 140.33, 139.28, 128.39, 127.83, 127.79, 127.45, 125.42, 125.05, 121.25, 120.13, 115.72, 115.70, 115.49, 115.46, 114.42. HRMS (QTOF+, m/z): calcd for $C_{31}H_{18}BrF_2N$ 521.0591, found 522.0669.

Synthesis of 10-(4-bromo-3,5-bis(3-bromophenoxy)phenyl)-10H-spiro[acridine-9,9'-fluor ene] (4): 10-(4-Bromo-3,5-difluorophenyl)-10*H*-spiro[acridine-9,9'-fluorene] (4.5 g, 8.61 mmol), 3-bromophenol (5.9 g, 34.4 mmol), and potassium carbonate (8.9 g, 51.6 mmol were dissolved in NMP (40 mL) under a nitrogen atmosphere. After stirring at 180 °C for 8 h, the reaction mixture was filtered through a pad of Florisil® using toluene as an eluent. After the solvent was evaporated, the crude product was washed with hexane. After purification, the product was obtained as a white solid (yield: 4.9 g, 70%). ^{1}H NMR (400 MHz, CD_2Cl_2) δ 7.85 (dd, *J* = 7.6, 0.9 Hz, 2H), 7.48 – 7.30 (m, 8H), 7.30 – 7.21 (m, 4H), 7.19 – 7.13 (m, 2H), 7.05 (ddd, *J* = 8.6, 7.2, 1.6 Hz, 2H), 7.00 (s, 2H), 6.67 – 6.56 (m, 2H), 6.49 (dd, *J* = 8.4, 1.1 Hz, 2H), 6.38 (td, *J* = 8.3, 1.6 Hz, 2H). ^{13}C

NMR (101 MHz, CD_2Cl_2) δ 157.27, 156.94, 156.29, 141.80, 140.48, 139.20, 131.34, 131.25, 128.32, 127.76, 127.74, 127.45, 127.39, 127.32, 125.40, 124.86, 123.09, 122.63, 121.84, 121.00, 120.78, 120.06, 118.17, 118.11, 117.27, 114.56, 114.34, 108.19. HRMS (QTOF+, m/z): calcd for $C_{43}H_{26}Br_3NO_2$ 824.9514, found 827.9590.

Synthesis of 10-(3,11-dibromo-5,9-dioxa-13b-boranaphtho[3,2,1-de]anthracen-7-yl)-10H-spiro[acridine-9,9'-fluorene] (M1): 10-(4-Bromo-3,5-bis(3-bromophenoxy)phenyl)-10*H*-spiro[acridine-9,9'-fluorene] (2.0 g, 2.41 mmol) in tert-butylbenzene (40.0 mL) at 0 ºC under nitrogen atmosphere was added dropwise a solution of *n*-BuLi (2.5 M solution in hexane, 1.06 mL, 2.65 mmol). The mixture was stirred at room temperature for 2 h. Then boron tribromide (0.3 mL, 3.01 mmol) was added at -20 ºC and stirred at room temperature for 1 h. Then *N*,*N*-diisopropylethylamine (0.57 g, 4.46 mmol) was added at 0 ºC and 120 ºC for 12 h, and then cooled to room temperature. The reaction mixture was filtered with a pad of Florisil® using toluene as an eluent. After the solvent was evaporated, the crude product was purified by column chromatography (eluent = *n*-hexane/toluene, 4:1). After purification, the product was obtained as a yellowness solid (yield: 0.73 g, 40%). ^{1}H NMR (400 MHz, Tetrachloroethane-d_2) δ 8.60 (d, J = 8.3 Hz, 2H), 7.95 – 7.89 (m, 4H), 7.69 (dd, J = 8.2, 1.8 Hz, 2H), 7.59 – 7.52 (m, 4H), 7.50 (t, J = 7.2 Hz, 2H), 7.39 (t, J = 7.4 Hz, 2H), 7.06 – 7.00 (m, 2H), 6.70 (t, J = 7.4 Hz, 2H), 6.64 – 6.50 (m, 4H). ^{13}C NMR (101 MHz, Tetrachloroethane-d_2) δ 160.98, 159.12, 156.38, 148.26, 141.03, 139.52, 135.53, 128.64, 128.47, 127.94, 127.81, 127.42, 127.14, 125.90, 125.48, 122.07, 121.26, 120.12, 115.00, 112.00. HRMS (QTOF+, m/z): calcd for $C_{43}H_{24}BBr_2NO_2$ 755.0267, found 757.0284.

Polymerization of PDBA-SAF-P8P: 10-(3,11-Dibromo-5,9-dioxa-13b-boranaphtho[3,2,1-*de*]anthracen-7-yl)-10*H*-spiro[acridine-9,9'-fluorene] (0.2000 g, 0.2641 mmol), 1,8-bis(4-(4,4,5,5-

tetramethyl-1,3,2-dioxaborolan-2-yl)phenyl)octane (0.1369 g, 0.2641 mmol), and potassium carbonate solution (3.0 mL, 2 mol/L in H_2O) with three drops of Aliquat 336 were dissolved in degassed toluene (9.0 mL), and then degassing with nitrogen for 15 min. SPhos (0.0032 g, 0.0079 mmol), and tris(dibenzylideneacetone)palladium(0) (0.0024 g, 0.0026 mmol) were added to the mixture, which was stirred for 5 days at 95 °C. After 5 days of reaction, bromobenzene (50 mg in 3 mL toluene) was added. After 8 hours, phenylboronic acid (100 mg in 3 mL toluene) was added. The reaction was cooled at room temperature and the polymer was precipitated in methanol, filtered through 0.45 μm nylon filter and washed on soxhlet apparatus with methanol, acetone, hexane and toluene. Then the toluene solution product was concentrated by rotary evaporation, precipitated into 300 mL methanol, filtered by vacuum to obtain the pure yellowness solid. (0.13 g, 60% yield). ^{1}H NMR (400 MHz, Tetrachloroethane-d_2) δ 9.16 (d, J = 8.0 Hz, 2H), 7.91 – 7.79 (m, 8H), 7.64 – 7.40 (m, 8H), 7.43 – 7.24 (m, 4H), 7.07 (dd, J = 20.2, 6.1 Hz, 6H), 6.82 – 6.34 (m, 8H), 2.87 – 2.58 (m, 4H), 1.71 (s, 6H), 1.37 (s, 6H). GPC (THF) M_n = 18000 g mol^{-1}, M_w = 22500 g mol^{-1}, PDI = 1.24 (against PS standard)).

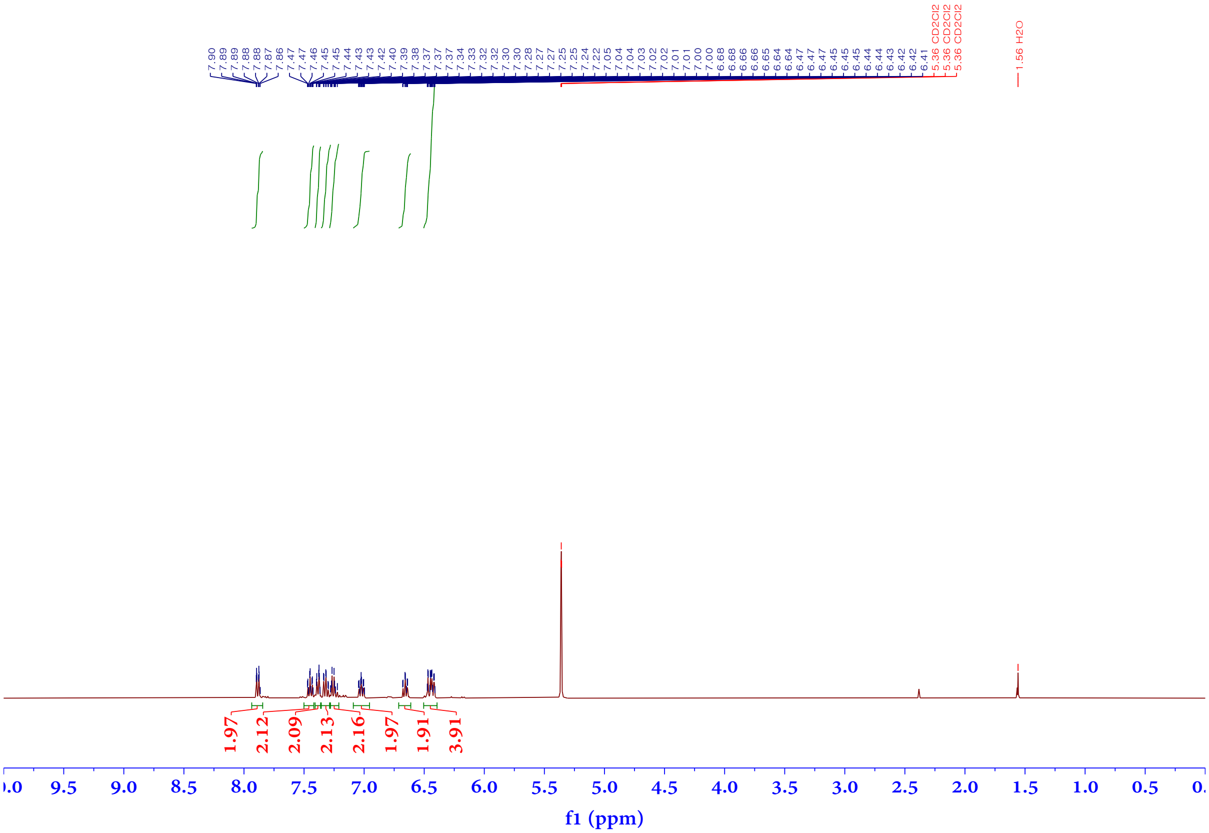

Fig. S1. 1H NMR spectrum of compound 3 in CD2Cl2.

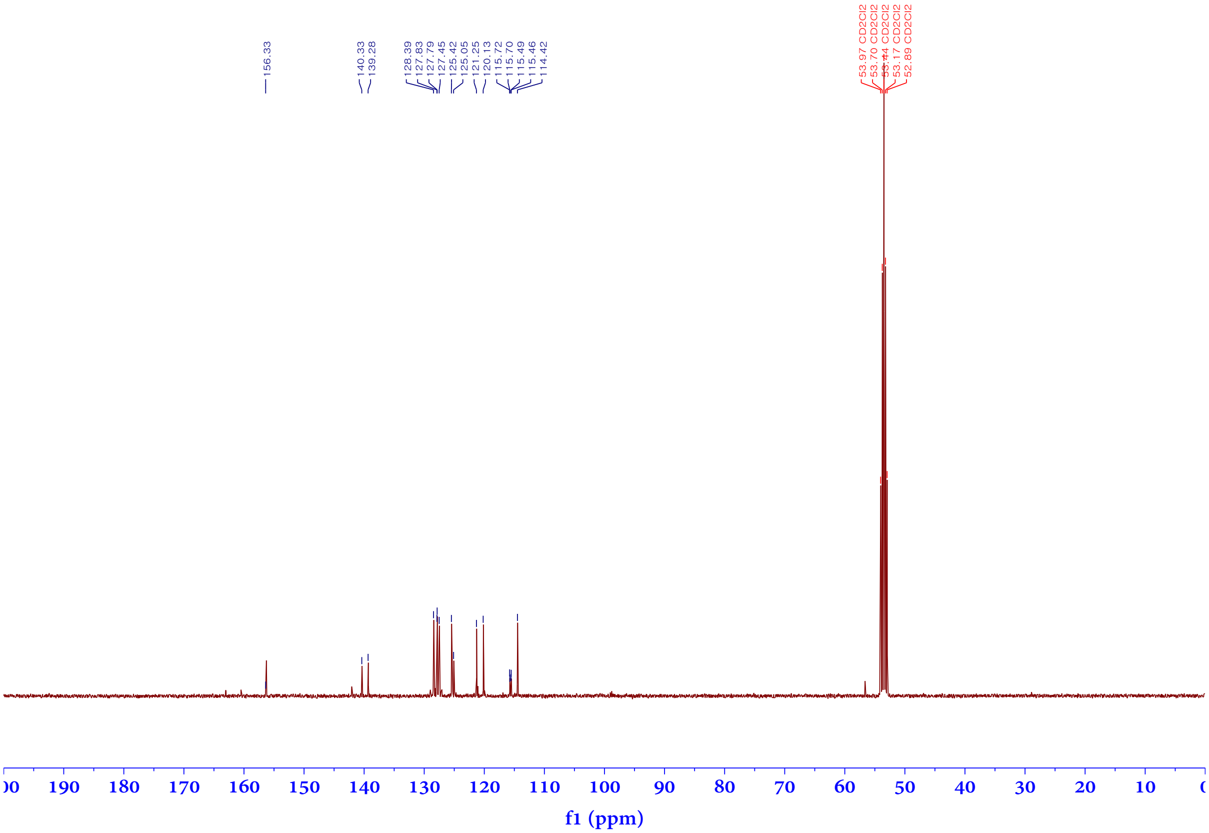

Fig. S2. ^{13}C **NMR spectrum of compound 3 in** CD_2Cl_2**.**

Elemental Composition

File Edit View Process Help

Single Mass Analysis

Tolerance = 20.0 PPM / DBE: min = 0.0, max = 120.0

Element prediction: Off

Monoisotopic Mass, Odd and Even Electron Ions

183 formula(e) evaluated with 7 results within limits (up to 10 closest results for each mass)

Elements Used:

C: 1-70 H: 1-60 N: 1-10 F: 1-4 Br: 1-1

Mass	Calc. Mass	mDa	PPM	DBE	Formula	C	H	N	F	Br
522.0669	522.0669	0.0	0.0	21.5	C31 H19 N F2 Br	31	19	1	2	1
	522.0667	0.2	0.4	18.0	C26 H18 N4 F3 Br	26	18	4	3	1
	522.0665	0.4	0.8	14.5	C21 H17 N7 F4 Br	21	17	7	4	1
	522.0728	-5.9	-11.3	18.0	C23 H17 N8 F2 Br	23	17	8	2	1
	522.0730	-6.1	-11.7	21.5	C28 H18 N5 F Br	28	18	5	1	1
	522.0604	6.5	12.5	22.0	C27 H16 N6 F Br	27	16	6	1	1
	522.0602	6.7	12.8	18.5	C22 H15 N9 F2 Br	22	15	9	2	1

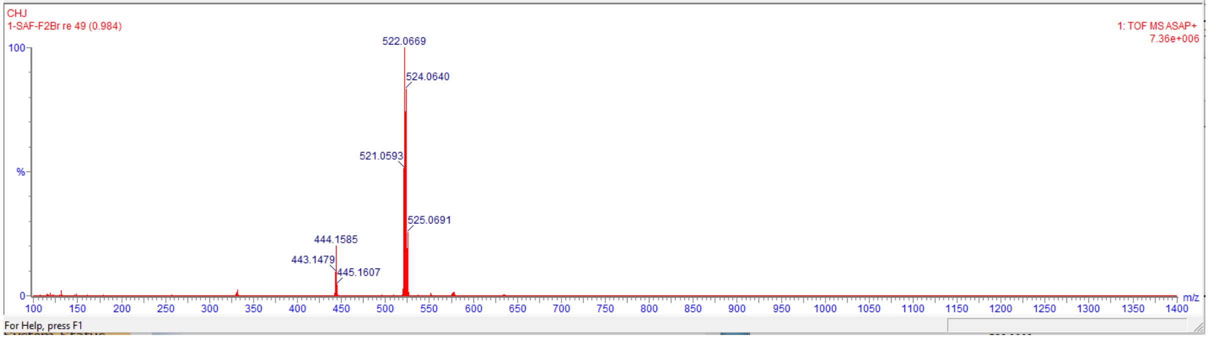

For Help, press F1

Fig. S3. QTOF+ Mass spectrum of compound 3.

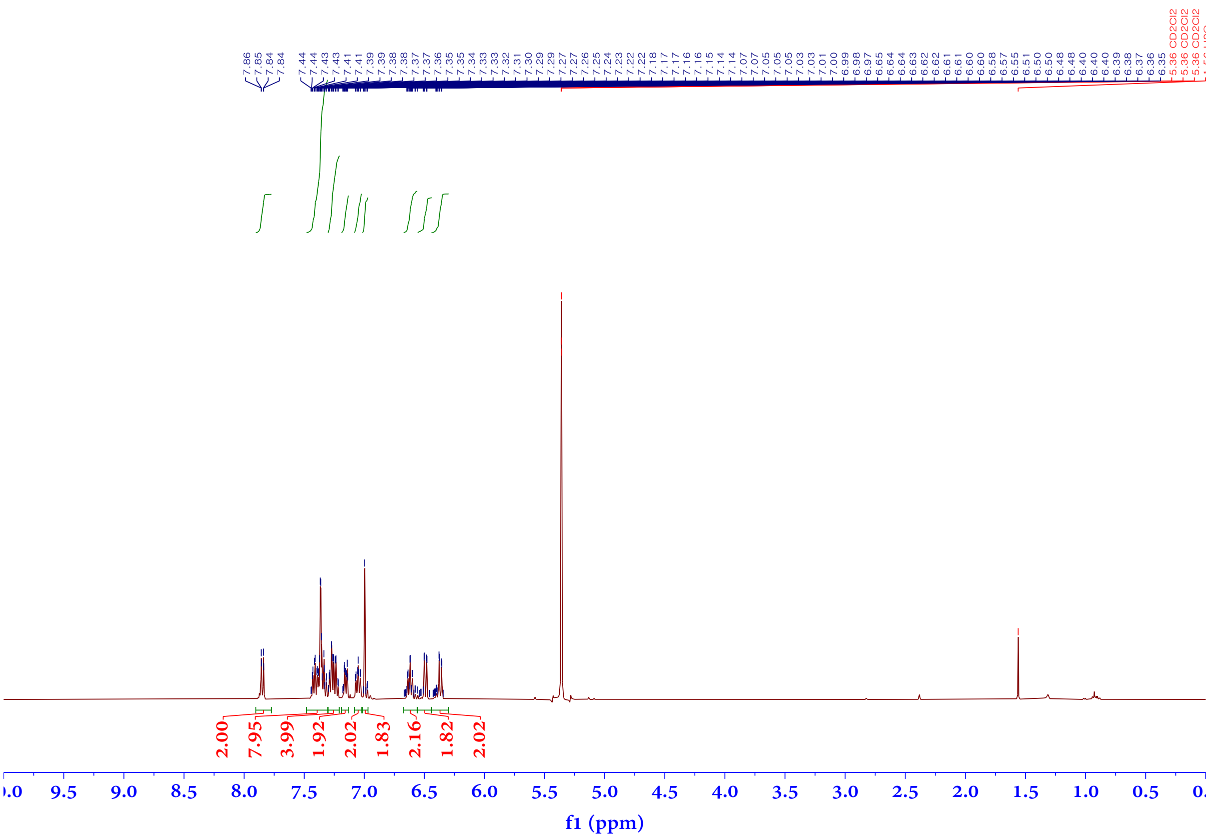

Fig. S4. ^{1}H NMR spectrum of compound 4 in CD_2Cl_2.

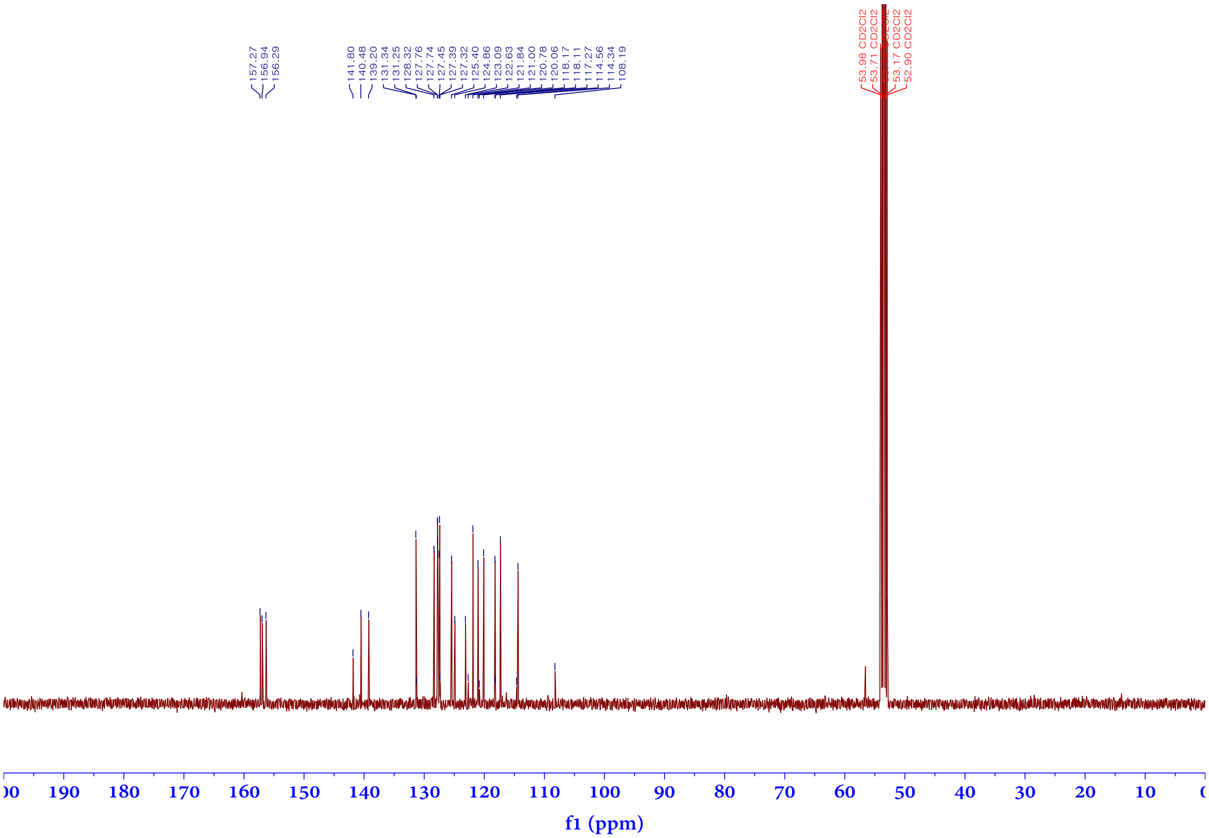

Fig. S5. ^{13}**C NMR spectrum of compound 4 in** CD_2Cl_2**.**

Elemental Composition

File Edit View Process Help

Single Mass Analysis

Tolerance = 20.0 PPM / DBE: min = 0.0, max = 120.0

Element prediction: Off

Monoisotopic Mass, Even Electron Ions

75 formula(e) evaluated with 5 results within limits (up to 5 closest results for each mass)

Elements Used:

C: 1-60 H: 1-50 N: 1-1 O: 1-2 79Br: 1-3 81Br: 1-3

Mass	Calc. Mass	mDa	PPM	DBE	Formula	C	H	N	O	79Br	81Br
827.9590	827.9571	1.9	2.3	29.5	C43 H27 N O2 79Br2 81Br	43	27	1	2	2	1
	827.9670	-8.0	-9.7	3.5	C29 H49 N O 79Br2 81Br3	29	49	1	1	2	3
	827.9510	8.0	9.7	18.5	C37 H36 N O 79Br3 81Br	37	36	1	1	3	1
	827.9483	10.7	12.9	3.5	C28 H47 N O2 79Br3 81Br2	28	47	1	2	3	2
	827.9731	-14.1	-17.0	14.5	C35 H40 N O2 79Br 81Br3	35	40	1	2	1	3

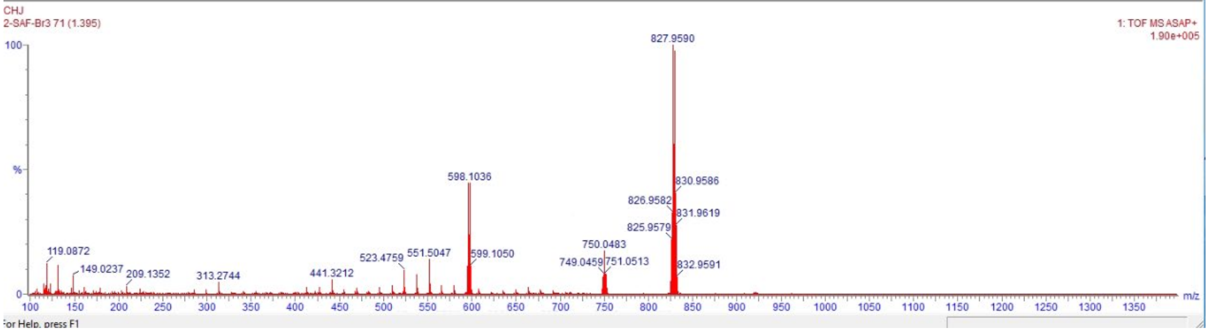

For Help, press F1

Fig. S6. QTOF+ Mass spectrum of compound 4.

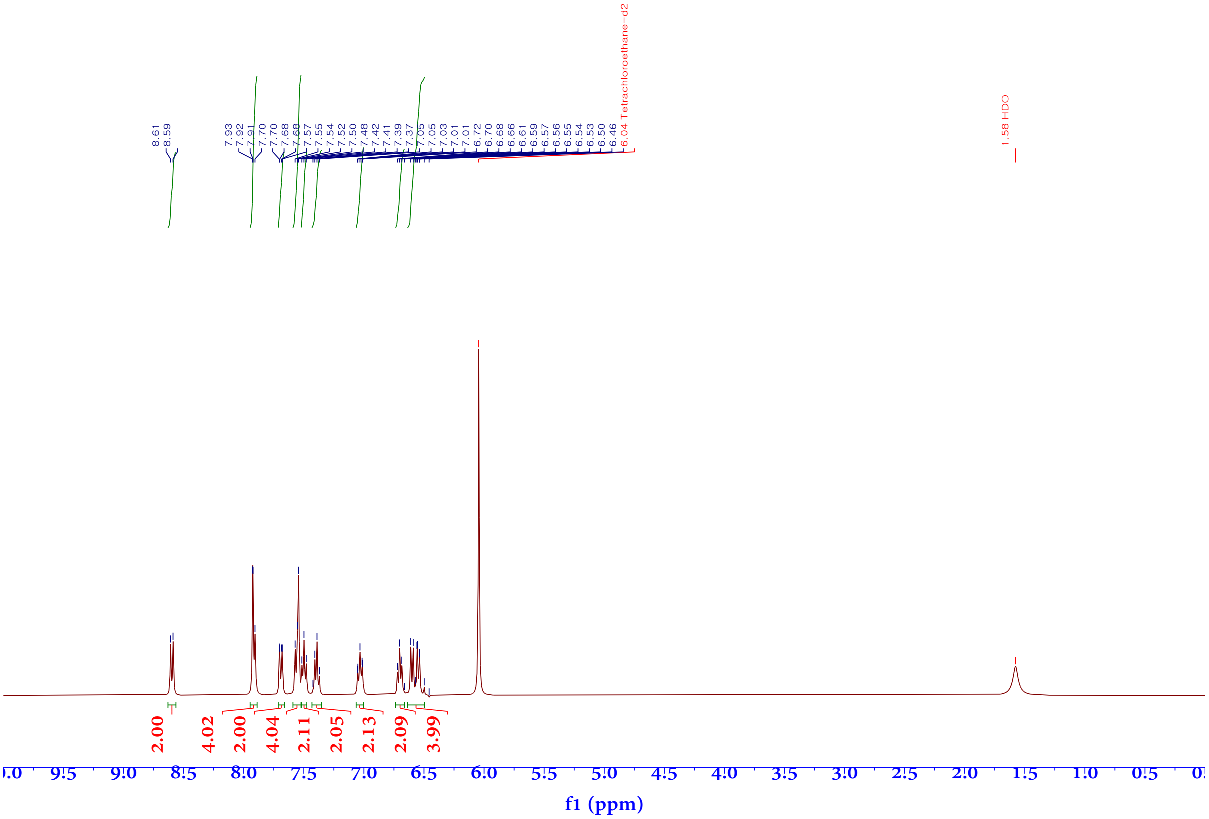

Fig. S7. ^{1}H NMR spectrum of M1 in $(CDCl_2)_2$ at 80 ºC.

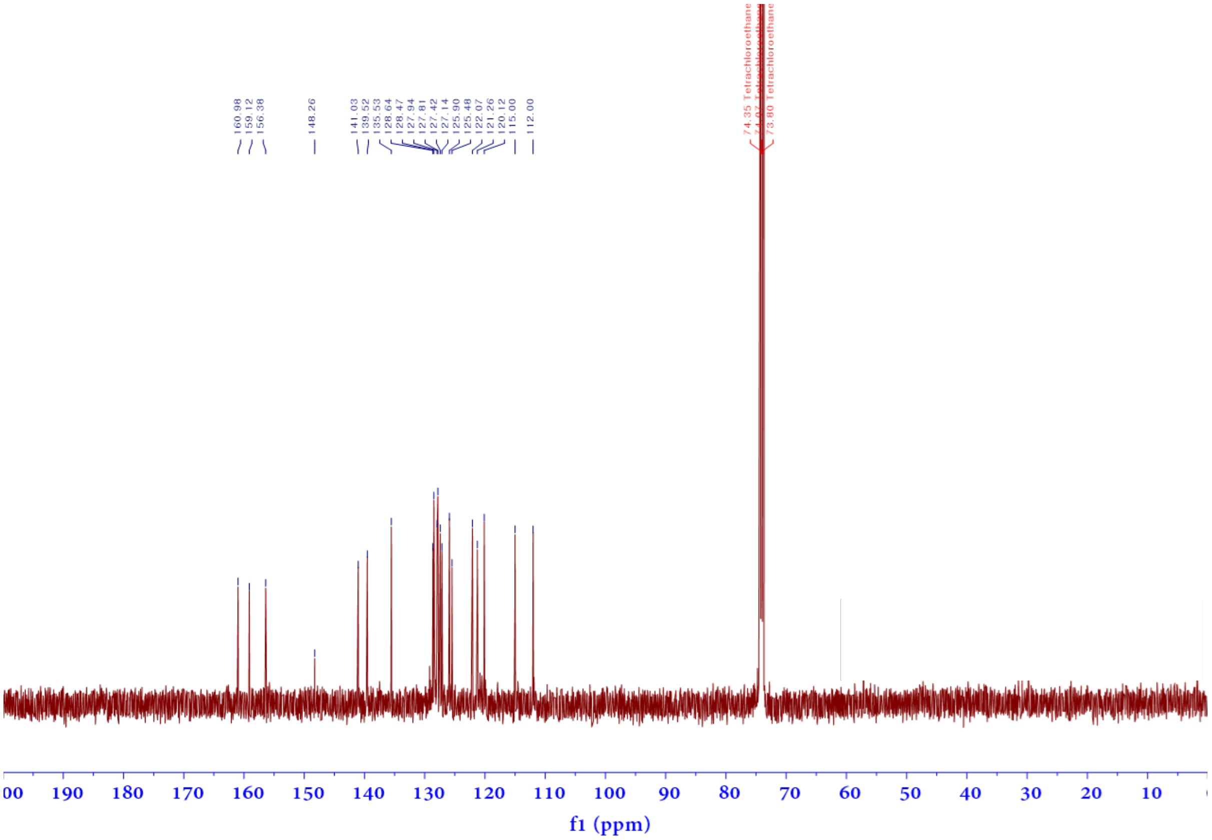

Fig. S8. ^{13}C NMR spectrum of M1 in $(CDCl_2)_2$ at 80 ºC.

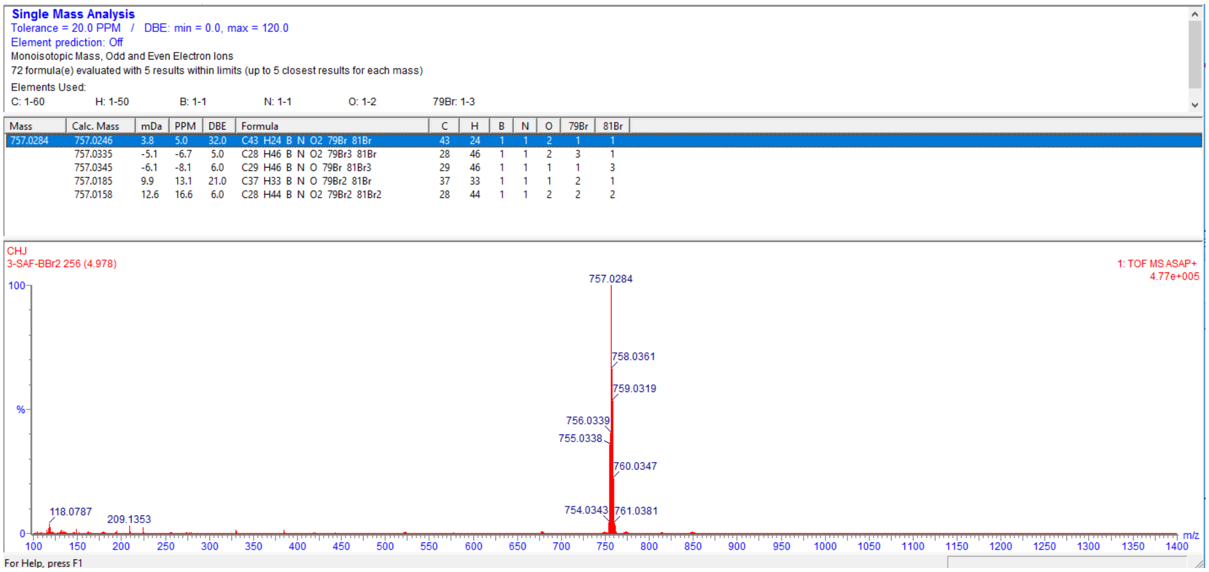

Fig. S9. QTOF+ Mass spectrum of M1.

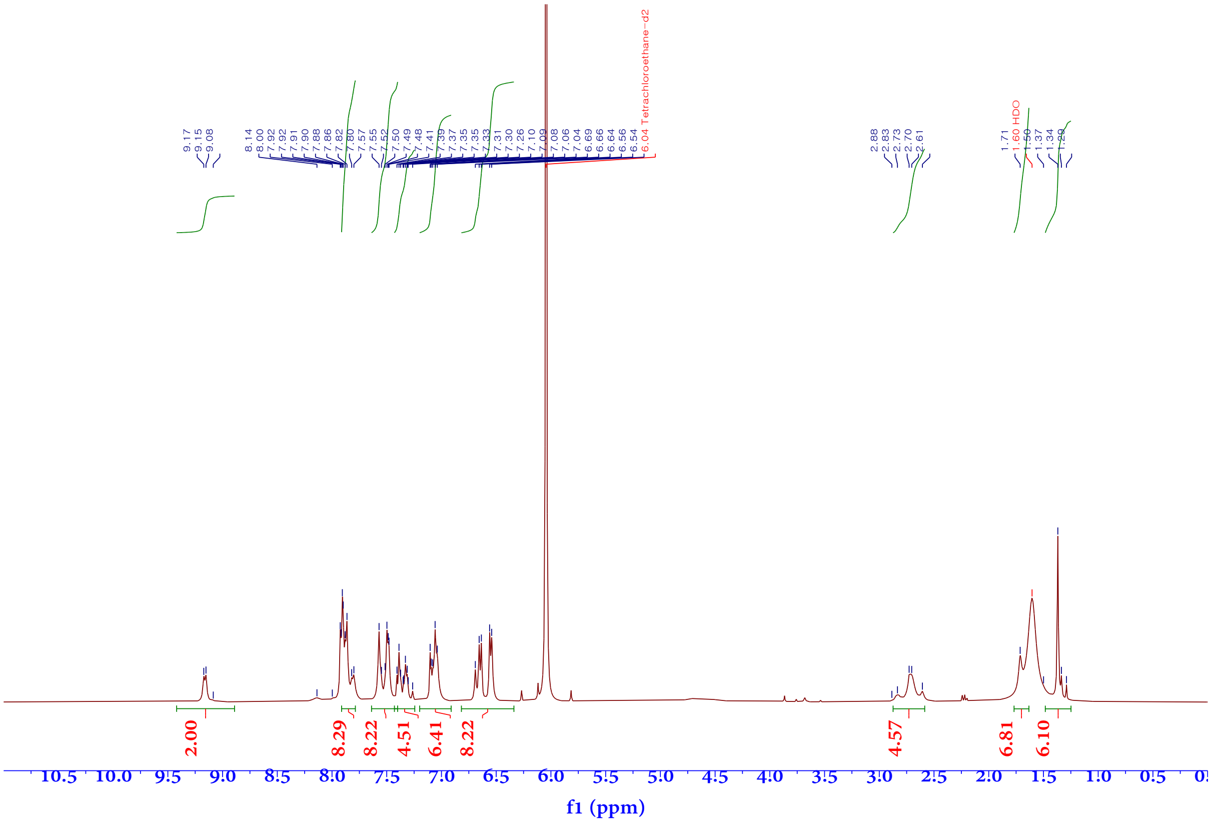

Fig. S10. ^{1}H NMR spectrum of PDBA-SAF-P8P in $(CDCl_2)_2$ at 80 ºC.

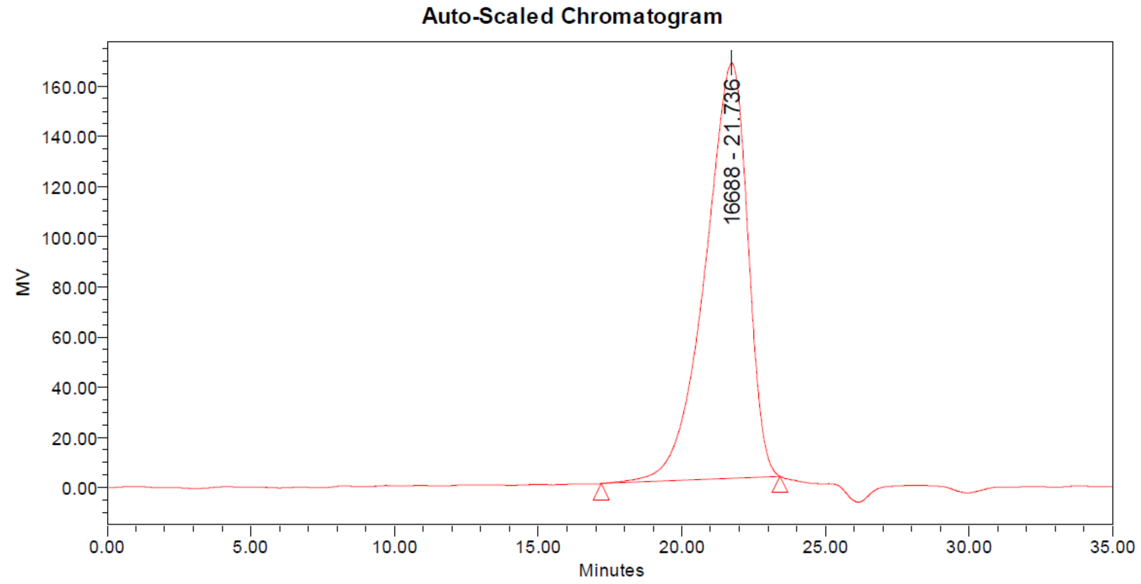

GPC Results

	Dist Name	Mn	Mw	MP	Mz	Mz+1	Mv	Polydispersity	MW Marker 1	MW Marker 2
1		18090	22513	16688	31412	52297		1.244497		

Fig. S11. Gel permeation chromatogram of PDBA-SAF-P8P obtained in THF solution.

The reaction was catalyzed by Pd(0) with SPhos as the ligand under nitrogen atmosphere at 95 °C for five days, followed by end-capping with bromobenzene and phenylboronic acid to minimize terminal defects. The resulting polymer was purified by Soxhlet extraction and obtained as a yellow solid with a yield of 60%. Gel permeation chromatography (GPC) revealed a number-average molecular weight (Mn) of 18,000 g mol−1 and a polydispersity index (PDI) of 1.24 against polystyrene standards.

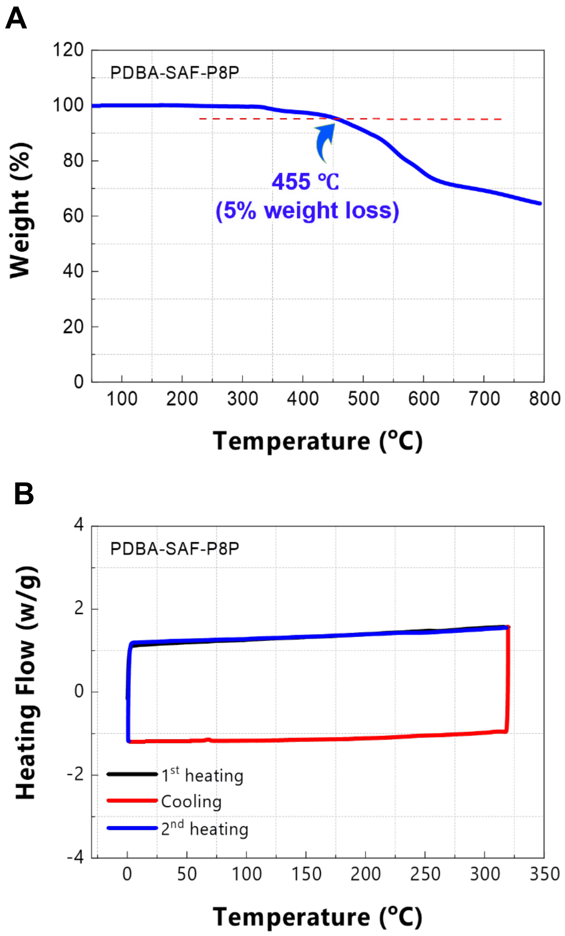

Fig. S12. Thermal properties of PDBA-SAF-P8P (**A**) Thermogravimetric analysis (TGA) and (**B**) differential scanning calorimeter (DSC) analysis of PDBA-SAF-P8P.

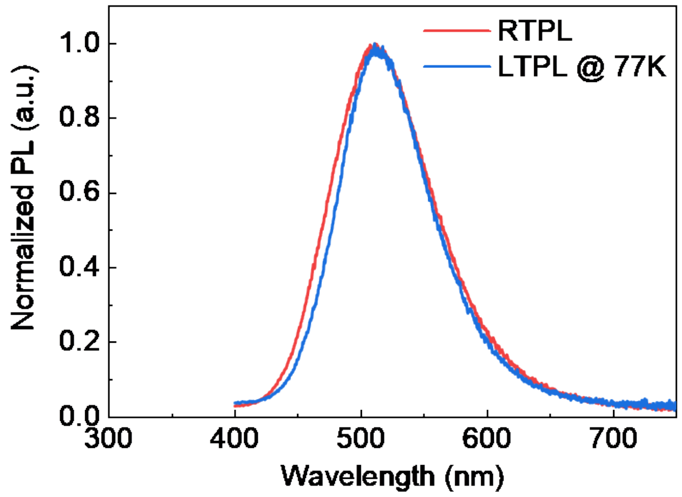

Fig. S13. Photoluminescence (PL), and low temperature (77 K) PL spectra of PDBA-SAF-P8P in a film state.

Low-temperature PL measurements (77 K) revealed a triplet emission onset at 2.77 eV, while the singlet energy level (S_1) was determined to be 2.84 eV, yielding a small singlet-triplet energy splitting (ΔE_{ST}) of 0.07 eV, which facilitates efficient reverse intersystem crossing (RISC) for TADF.

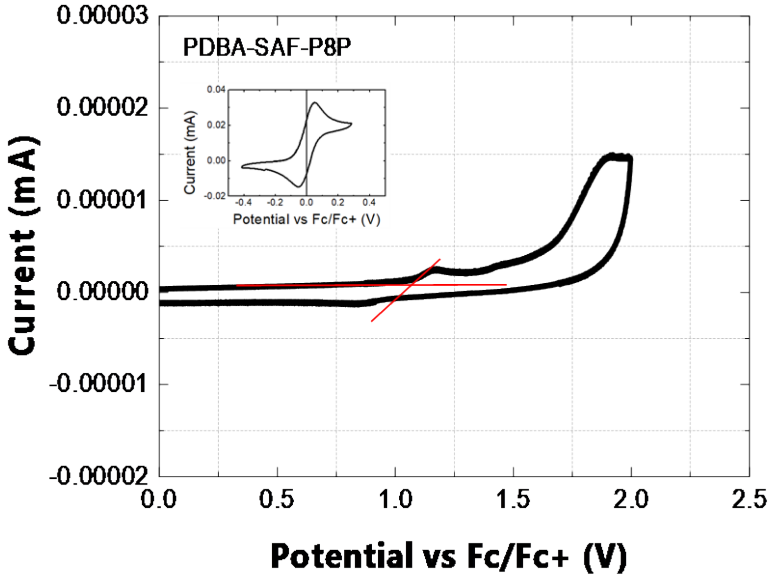

Fig. S14. Cyclic voltammetry (CV) analysis of PDBA-SAF-P8P.

Table S1. Photophysical & electrochemical properties of PDBA-SAF-P8P.

	λ_{Abs} [nm]	λ_{PL} / FWHM [a] [nm]	Stoke shift [nm]	S_1 / T_1 [b] [eV]	ΔE_{ST} [c] [eV]	HOMO / LUMO [d] [eV]	Band gap [e] [eV]
**PDBA-SAF-P8P**	310, 375	465 / 58	90	2.84 / 2.77	0.07	-5.53 / -2.46	3.07

[a]Photoluminescence maximum emission measured in toluene solution. [b]Singlet energy obtained from the onset of the RTPL/triplet energy obtained from the onset of the LTPL in a film state. [c]$\triangle E_{ST} = S_1 - T_1$. [d]The HOMO energy level was measured by CV according to $E_{HOMO} = -(4.42 + E_{onset}^{ox})$ eV and LUMO level was caculated from the HOMO level and optical band gap. [e]The optical band gap was measured by absorption onset (Eg = 1240 / λ) by UV-vis spectroscopy.

Table S2. Photophysical properties and rate constants from the emission decay of PDBA-SAF-P8P in film.

	ϕ_{PL} [a]	ϕ_p [b]	ϕ_d [b]	τ_p [c] [ns]	τ_d [c] [μs]	k_r [d] [10^7 s^{-1}]	k_{ISC} [e] [10^7 s^{-1}]	k_{RISC} [f] [10^6 s^{-1}]
**PDBA-SAF-P8P**	0.7	0.39	0.31	31.4	1.21	1.27	1.94	1.08

[a]Photoluminescence quantum yield (PLQY) in film, excited at 310 nm. [b]The contribution of prompt fluorescence (ϕ_p) component and delayed fluorescence (ϕ_d) component to the PLQY for the thin film. [c]The prompt fluorescence (τ_p) and delayed fluorescence (τ_d) lifetime. [d]The radiative decay rate constants. [e]The rate constants of intersystem crossing. [f]The rate constants of reverse intersystem crossing.

The rate constants were determined using the following equations (Equations S1 to S8) *(11, 53, 54).*

$$k_p = \frac{1}{\tau_p} \tag{S1}$$

$$k_d = \frac{1}{\tau_d} \tag{S2}$$

The k_{RISC} is calculated by assuming $k_{nr}^S << k_r^S$, k_{ISC} and $k_p >> k_d$, the corresponding rate constants can be calculated with the following equations:

$$k_r^s = \phi_p k_p + \phi_d k_d \approx \phi_p k_p \tag{S3}$$

$$k_{ISC} = k_p(1 - \phi_p) \tag{S4}$$

$$k_{RISC} = \frac{k_p k_d \phi_d}{k_{ISC} \phi_p} \tag{S5}$$

FRET efficiency and k_{FRET} can be estimated from τ_p^{HF} and $\tau_p^{sensitizer}$, which are the prompt lifetimes of the sensitizer emission quenched by the terminal emitter in the hyperfluorescence film (host:polymer sensitizer:MR-TADF emitter) and the sensitizer in the sensitizer film (host:polymer sensitiser),respectively. k_{DET} is then estimated with the prompt and delayed lifetime of the MR-

TADF film and the sensitizer film, the corresponding rate constants can be calculated the following equations:

$$\eta_{FRET} = 1 - \frac{\tau_p^{HF}}{\tau_p^{sensitizer}} \tag{S6}$$

$$k_{FRET} = \frac{\eta_{FRET}}{\tau_p^{HF}} \tag{S7}$$

$$k_{DET} = \frac{1}{\tau_d^{MR-TADF}} - \frac{1}{\tau_d^{sensitizer}} + k_{ISC}k_{RISC}(\tau_p^{MR-TADF} - \tau_p^{sensitizer}) \tag{S8}$$

II. Additional Figures

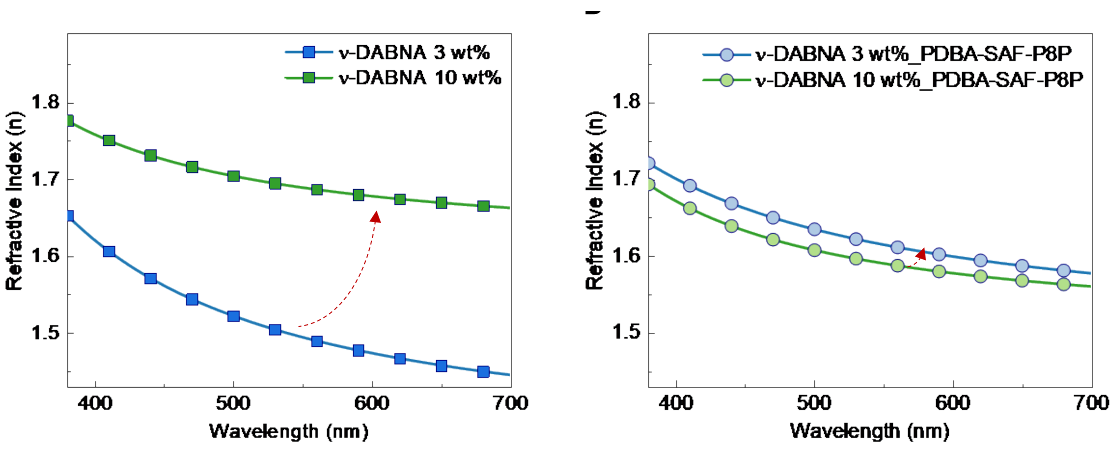

Fig. S15. Refractive index of solution-processed films. (**A**) mCP:DBFPO:ν-DABNA films with 3 and 10 wt% ν-DABNA, and (**B**) corresponding films incorporating 33 wt% PDBA-SAF-P8P.

We examined the refractive index (*n*) evolution as an optical proxy for molecular packing and aggregation in the solution-processed films. Increasing the ν-DABNA concentration from 3 wt% to 10 wt% in the small-molecule host matrix resulted in a noticeable increase in n across the measured spectral range (from 1.52 to 1.71 at 500 nm). This trend is commonly associated with increased molecular packing density and enhanced intermolecular interactions (*20*), which are consistent with aggregation of planar MR-TADF emitters in solution-processed films.

In contrast, when 33 wt% of the polymer sensitizer PDBA-SAF-P8P (relative to the host matrix) was incorporated, the refractive index remained nearly unchanged even at high ν-DABNA concentrations. This behavior indicates that the polymer effectively disrupts close molecular packing of the MR-TADF emitters, thereby suppressing aggregation and phase segregation.

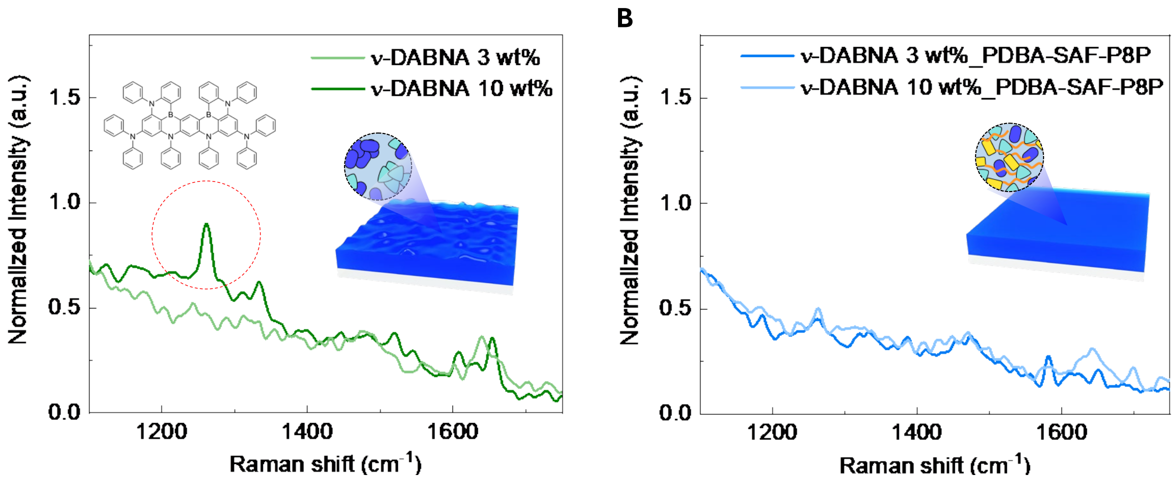

Fig. S16. Raman spectra of solution-processed films. (**A**) mCP:DBFPO:ν-DABNA films with 3 and 10 wt% ν-DABNA, and (**B**) corresponding films incorporating 33 wt% PDBA-SAF-P8P.

Raman spectroscopy provides further molecular-level evidence for aggregation suppression by the polymer sensitizer. In the small-molecule host system without polymer, increasing the ν-DABNA concentration to 10 wt% induces the emergence of a pronounced Raman feature at ~1260 cm^{-1}, which is absent or weak at lower dopant concentrations. This vibration is associated with phenyl-ring-related C-N/C-C stretching modes and is known to be sensitive to intermolecular π-π interactions (*21, 22*). The appearance and enhancement of this peak at high ν-DABNA loading therefore indicate strengthened intermolecular coupling and aggregation of the MR-TADF emitter.

By contrast, when 33 wt% PDBA-SAF-P8P is incorporated, the Raman spectrum of the 10 wt% ν-DABNA film no longer exhibits the distinct ~1260 cm^{-1} feature. The suppression of this aggregation-related vibrational signature suggests that the polymer sensitizer effectively disrupts close π-π stacking between adjacent ν-DABNA molecules, leading to a more homogeneous molecular environment.

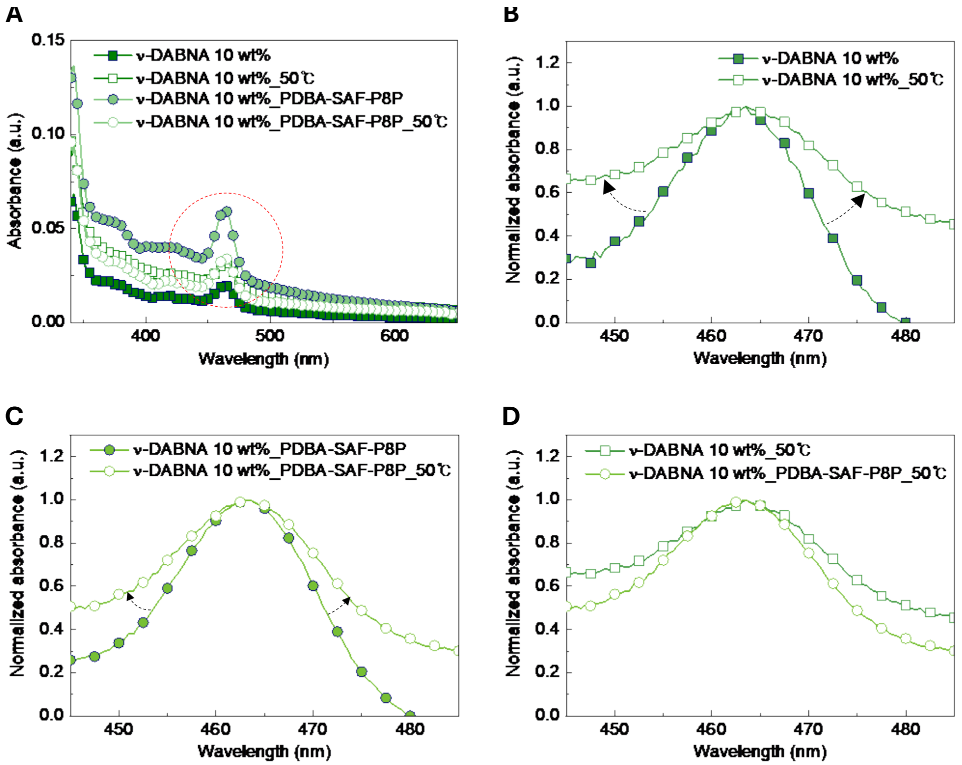

Fig. S17. UV-vis absorption spectra of solution-processed Films. (**A**) Absolute absorption spectra before and after thermal annealing at 50 °C. (**B-D**) Normalized absorption spectra highlighting annealing-induced spectral broadening and tailing effects.

In solution-processed EMLs, post-deposition thermal annealing is commonly required to remove residual solvent and relax the film. However, such thermal treatment can also promote molecular rearrangement and packing evolution, which may trigger or exacerbate aggregation in planar small-molecule emitters (*20*). Consistent with this, the 10 wt% ν-DABNA film shows a clear annealing-induced spectral broadening even at low temperature of 50℃ reflected by the emergence/enhancement of a broadened absorption feature and tailing behavior.

In contrast, when 33wt% PDBA-SAF-P8P is incorporated together with ν-DABNA, the spectral change upon annealing is markedly suppressed: the absorption profile remains comparatively

stable, indicating that the polymer matrix effectively suppresses aggregation and stabilizes molecular packing against thermally induced reorganization, thereby improving the morphological robustness.

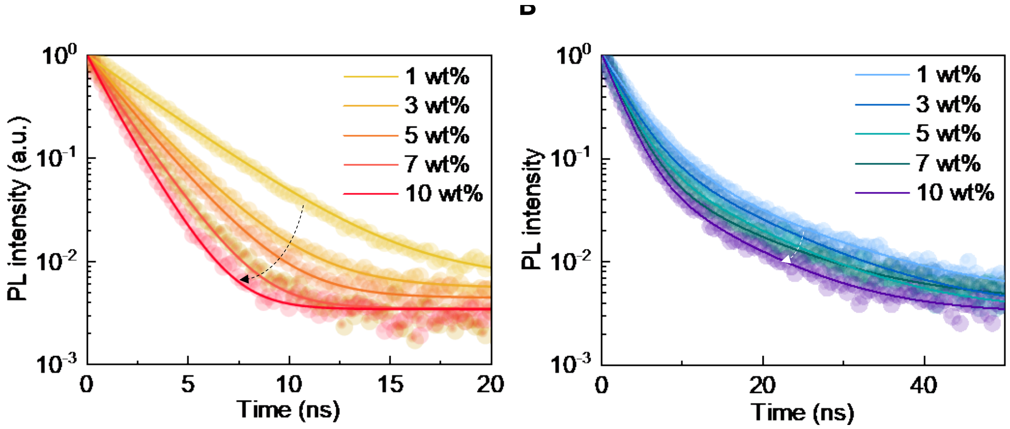

Fig. S18. Time-resolved photoluminescence (TRPL) spectra of solution-processed mCP:DBFPO:ν-DABNA films. (**A**) Films with 33 wt% polymer sensitizer and (**B**) without polymer sensitizer.

TRPL measurements further corroborate the stabilizing role of the polymer sensitizer on exciton dynamics. In the small-molecule films without polymer, increasing the ν-DABNA concentration leads to a pronounced acceleration of PL decay, with the lifetime decreasing from 3.13 ns at 1 wt% to 1.19 ns at 10 wt%. This sharp lifetime shortening is indicative of strong aggregation-induced exciton quenching and enhanced Dexter-type energy transfer between closely packed MR-TADF molecules.

By contrast, in the films containing 33 wt% PDBA-SAF-P8P, the PL lifetime decreases more gradually to 2.81 ns with 10 wt% ν-DABNA, corresponding to a significantly smaller reduction. These results demonstrate that the polymer matrix effectively suppresses aggregation-induced nonradiative decay pathways and mitigates Dexter-type exciton quenching, thereby stabilizing exciton lifetimes even at elevated emitter concentrations.

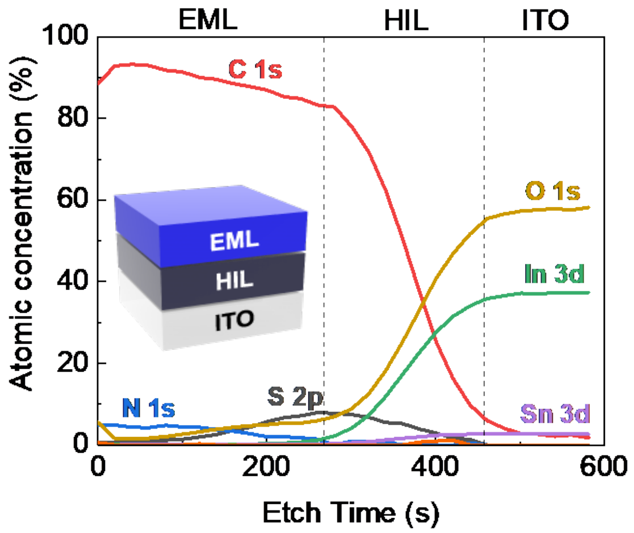

Fig. S19. Depth profile of x-ray photoelectron spectroscopy (XPS) measurements of atomic concentrations as a function of etch time for ITO/PEDOT:PSS/EML.

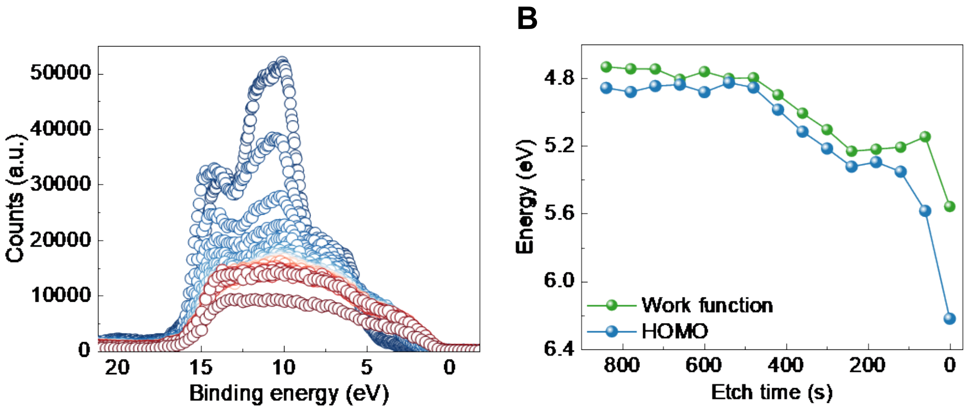

Fig. S20. Depth-resolved electronic structure of f-PEDOT:PSS. (**A**) UPS depth profiles and (**B**) corresponding energy structure as a function of etch time for *f*-PEDOT:PSS.

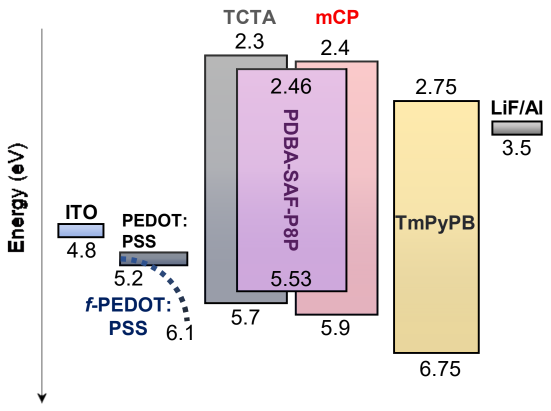

Fig. S21. Energy level diagram of solution-processed organic light-emitting diodes (SOLEDs) using PDBA-SAF-P8P as emitter with TCTA or mCP single-host.

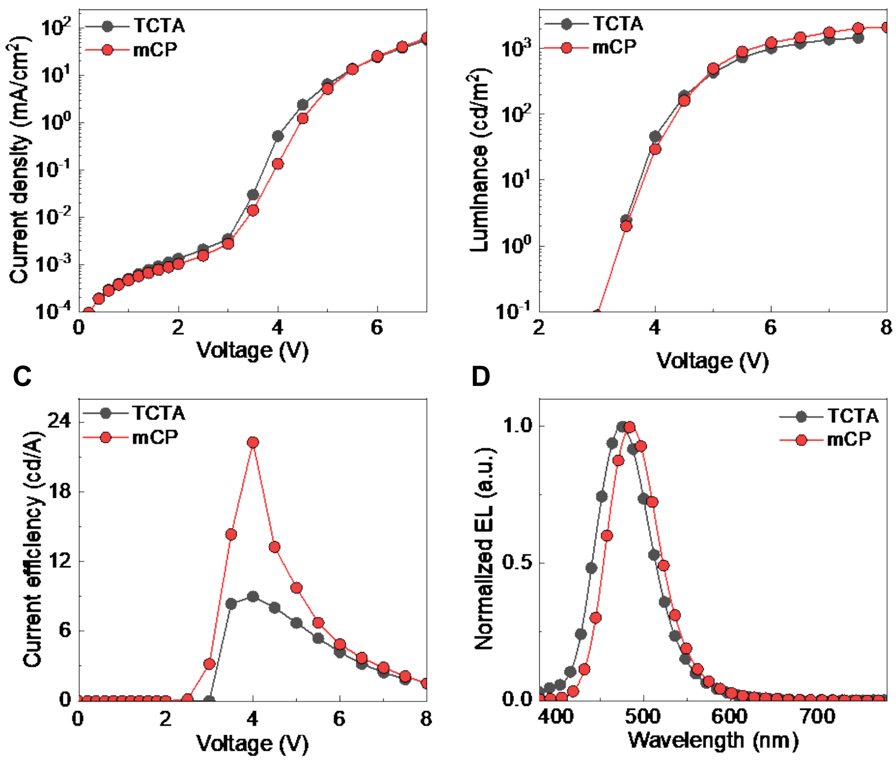

Fig. S22. Host-dependent device performance of sensitizer-only TADF OLEDs. (**A**) Current density versus voltage, (**B**) luminance versus voltage, (**C**) Current efficiency versus voltage characteristics and (**D**) Normalized electroluminescence (EL) spectra of SOLEDs using *f*-PEDOT:PSS, and PDBA-SAF-P8P as emitter with TCTA or mCP single-host.

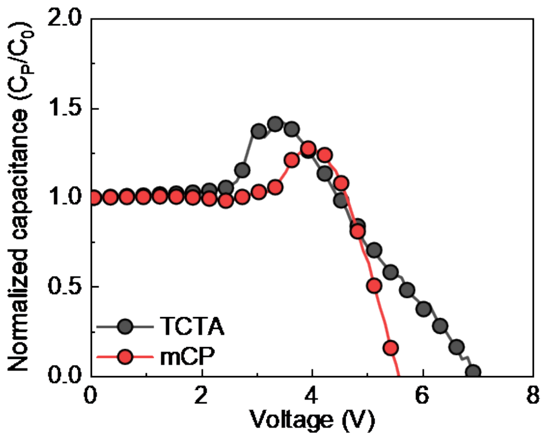

Fig. S23. Normalized capacitance versus voltage characteristics of SOLEDs using *f*-PEDOT:PSS, and PDBA-SAF-P8P as emitter with TCTA or mCP single-host.

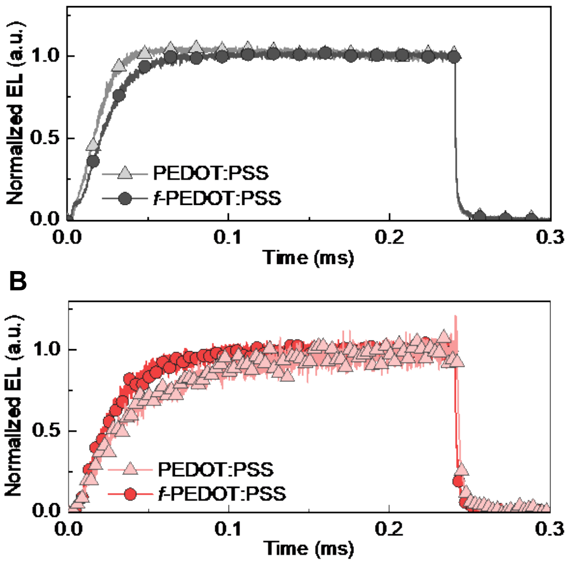

Fig. S24. Transient electroluminescence (EL) characteristics. (**A**) TCTA or (**B**) mCP single-host with PEDOT:PSS and *f*-PEDOT:PSS.

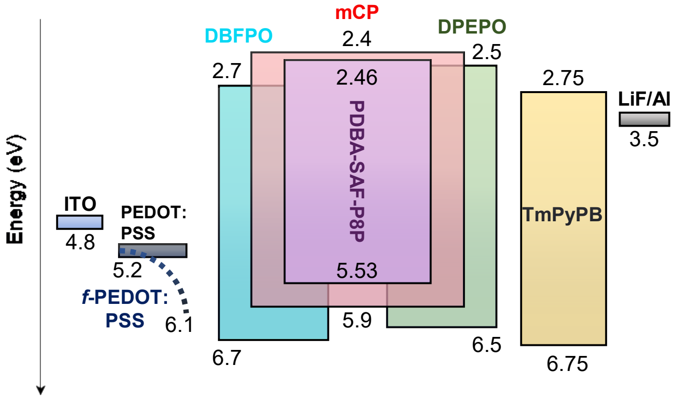

Fig. S25. Energy level diagram of SOLEDs using PDBA-SAF-P8P as emitter with mCP, DBFPO, and DPEPO host.

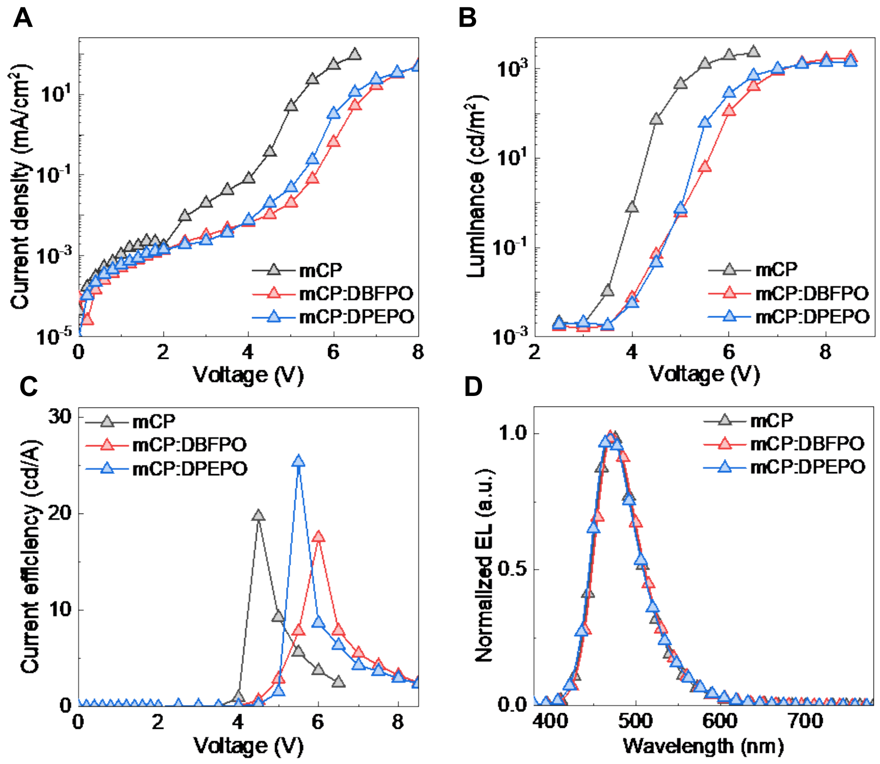

Fig. S26. Device performance of SOLEDs using PEDOT:PSS. (**A**) Current density versus voltage, (**B**) luminance versus voltage, (**C**) Current efficiency versus voltage characteristics and (**D**) Normalized electroluminescence (EL) spectra.

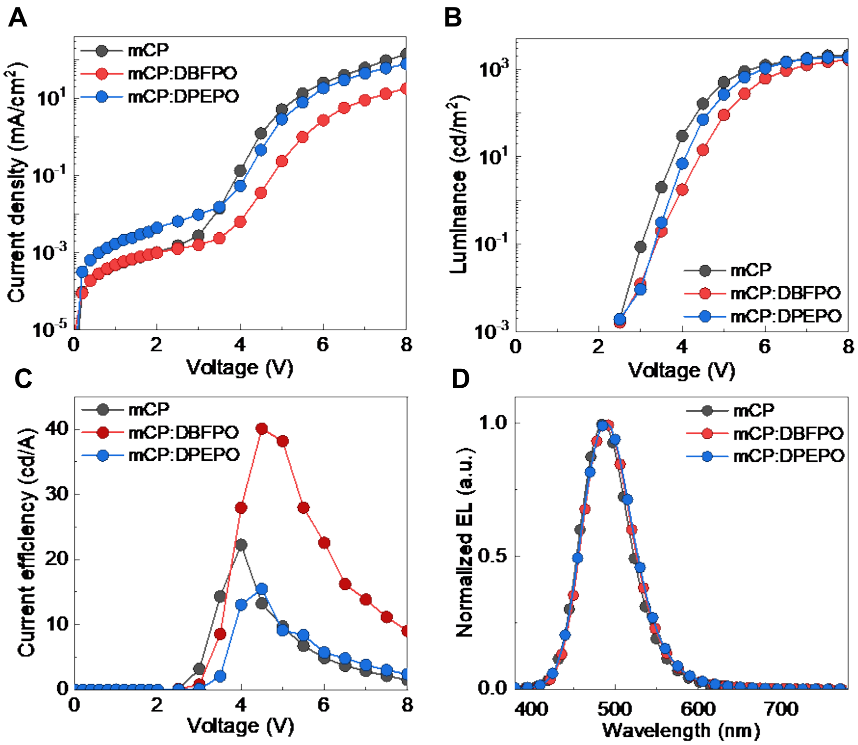

Fig. S27. Device performance of SOLEDs using *f*-PEDOT:PSS. (**A**) Current density versus voltage, (**B**) luminance versus voltage, (**C**) Current efficiency versus voltage characteristics and (**D**) Normalized electroluminescence (EL) spectra .

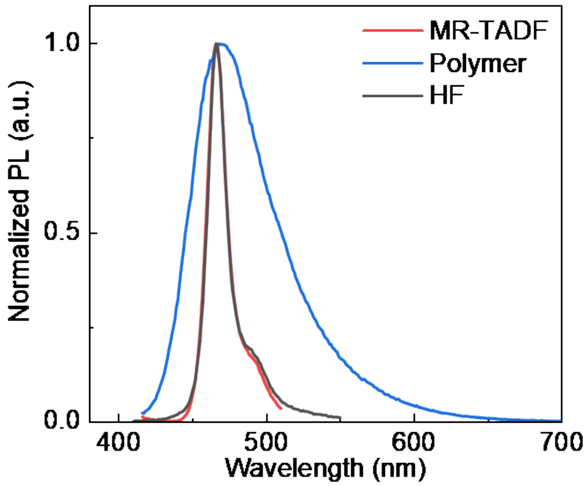

Fig. S28. Normalized PL spectra of host (mCP:DBFPO), MR-TADF (mCP:DBFPO:ν-DABNA), polymer (mCP:DBFPO:PDBA-SAF-P8P) and HF (mCP:DBFPO:PDBA-SAF-P8P:ν-DABNA) in THF solution.

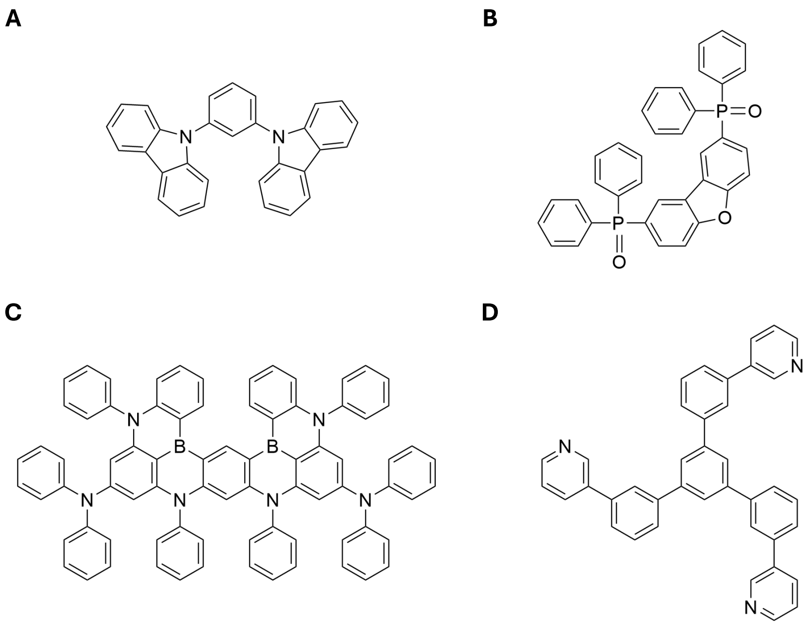

Fig. S29. Molecular structures of small-molecule materials. (**A**) mCP, (**B**) DBFPO, (**C**) ν-DABNA, and (**D**) TmPyPB

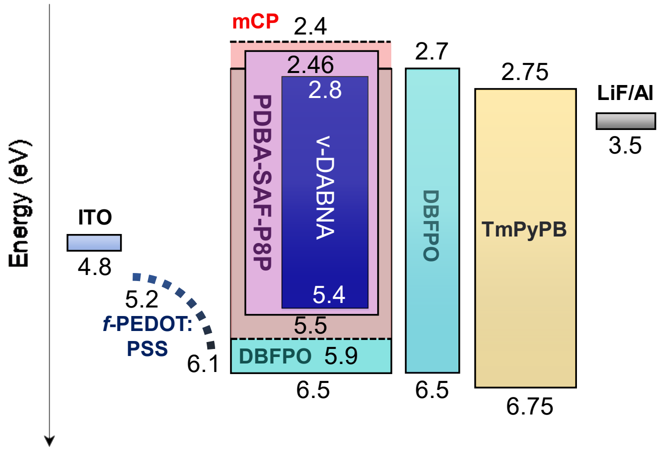

Fig. S30. Energy level diagram of SOLEDs using hyperfluorescent EML with optimized architecture.

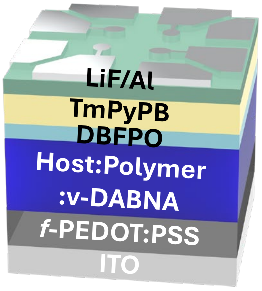

Fig. S31. Device structure of SOLEDs using hyperfluorescent EML with optimized architecture.

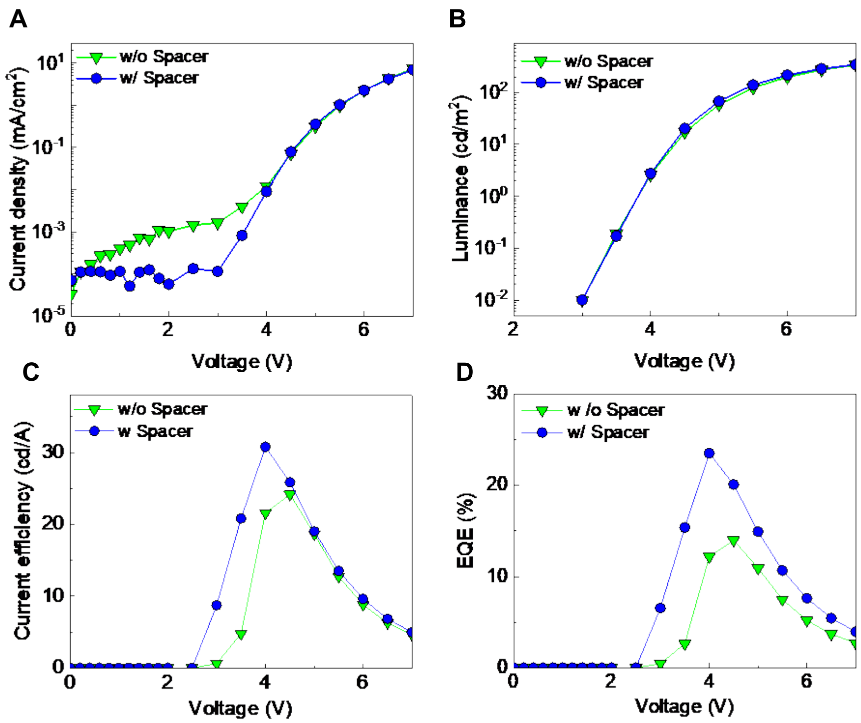

Fig. S32. Effect of a DBFPO spacer on HF SOLED performance. (**A**) Current density versus voltage, (**B**) luminance versus voltage, (**C**) Current efficiency versus voltage, and (**D**) EQE versus voltage characteristics.

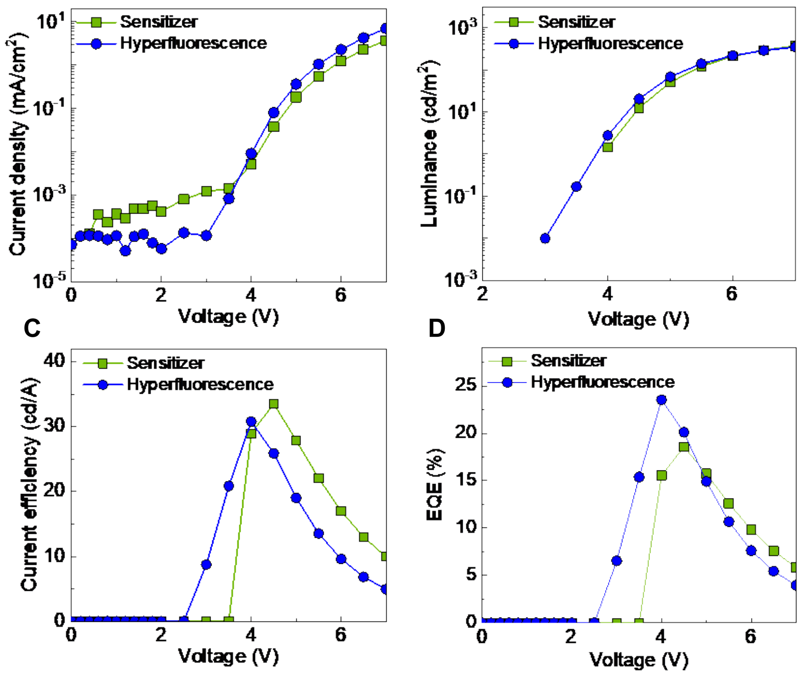

Fig. S33. Device characteristics of host:polymer and hyperfluorescent SOLEDs. (**A**) Current density versus voltage, (**B**) luminance versus voltage, (**C**) Current efficiency versus voltage, and (**D**) EQE versus voltage characteristics.

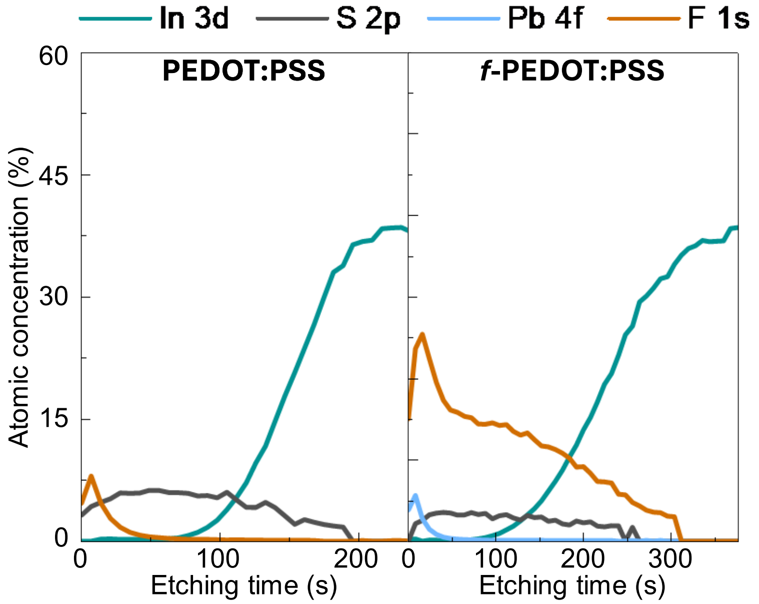

Fig. S34. XPS depth profiles of In, S, Pb, and F of ITO/HIL/EML stack for PEDOT:PSS and PEDOT:PSS:PFSA (*f*-PEDOT:PSS).

While *f*-PEDOT:PSS significantly enhances interfacial stability and provides improvements in hole injection by forming a PFSA-enriched surface via self-organization, it also introduces a limitation. Specifically, at higher fluorine content, the surface-segregated PFSA chains form a thick insulating top layer that increases the tunneling barrier between the anode and the EML.

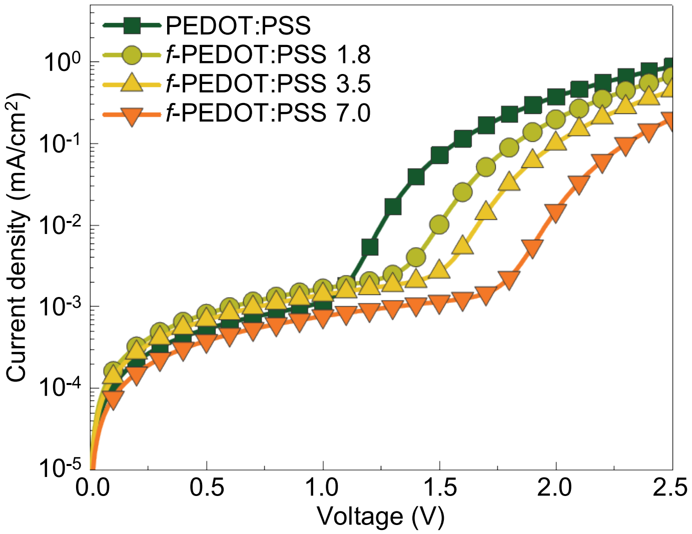

Fig. S35. Current density versus voltage characteristics of hole-only devices with various PFSA concentration.

This can limit efficient hole injection and consequently increases the turn-on voltage of OLEDs. As the PFSA content in the HIL increases, the threshold voltage gradually rises, reflecting hindered hole injection from the HIL into the EML. Moreover, the PFSA chains do not participate in any strong chemical coordination at the interface, which results in a random dipole distribution and prevents the formation of a coherent vacuum level shift at the surface. These factors ultimately cap the injection performance of *f*-PEDOT:PSS and motivate the need for additional interfacial engineering.

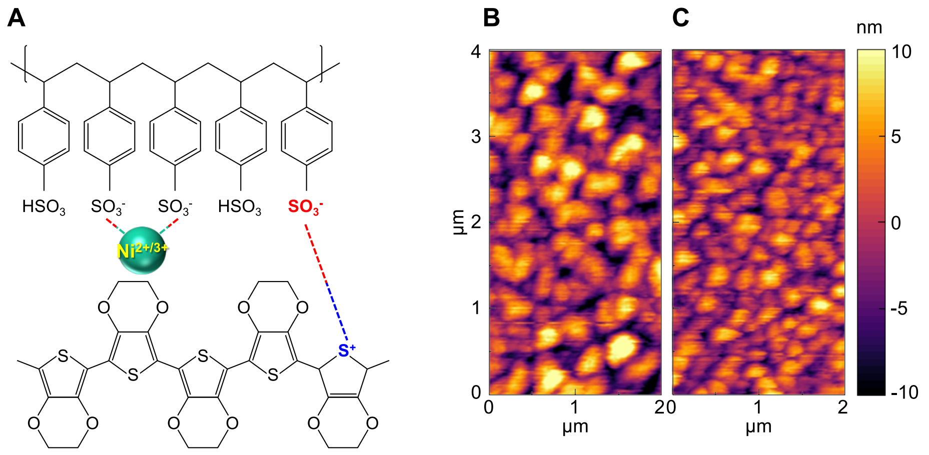

Fig. S36. NiOx-induced de-doping and morphology of PEDOT:PSS-based HILs. (**A**) Schematic illustration of de-doping of PEDOT induced by cation-anion pair complexes between Ni^{2+}, Ni^{3+} ions and sulfonates in PSS. AFM images of (**B**) *f*-PEDOT:PSS, (**C**) and *th*-HIL.

To overcome the thick tunneling barrier induced by the insulating PFSA surface layer and to further enhance energetic alignment at the interface, we additionally incorporated NiO_x into the f-PEDOT:PSS. The incorporation of Ni^{2+} ions enables strong ionic coordination with the sulfonate groups of both PFSA and PSS, leading to the formation of Ni-sulfonate complexes that fundamentally modify the self-organization behavior during film formation. Atomic force microscopy (AFM) analysis showed that the resulting ternary hybrid HIL (*th*-HIL) exhibits a significantly finer and more uniform surface morphology compared to *f*-PEDOT:PSS, indicating improved topographical and energetic homogeneity. This morphological refinement is attributed to the Ni cation-mediated suppression of lateral phase coarsening within the PFSA/PEDOT:PSS matrix.

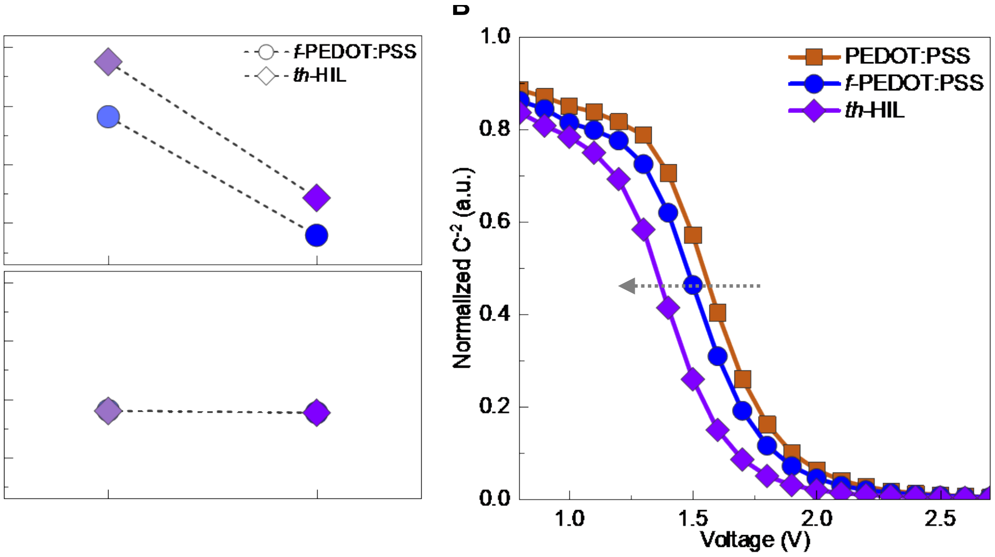

Fig. S37. Surface energetics and built-in potential of hole injection layers. (**A**) Dispersive and polar components of surface energy derived from contact angle, and (**B**) Mott-Schottky analysis of HODs showing built-in potential (V_{bi}) for PEDOT:PSS, *f*-PEDOT:PSS , and *th*-HIL.

The fine-grained surface of *th*-HIL further promotes more coherent ionic coordination between PFSA sulfonate groups and Ni^{2+} ions, ultimately giving rise to a well-aligned interfacial dipole layer at the HIL surface.

The ternary hybrid HIL (*th*-HIL) design incorporating NiO_x provides synergistic benefits by simultaneously improving energy-level alignment, interfacial morphology, and exciton management. The Ni-sulfonate complexation promotes coherent dipole alignment at the top interface, which induces a net vacuum level shift and reduces the energetic barrier for hole injection into the EML.

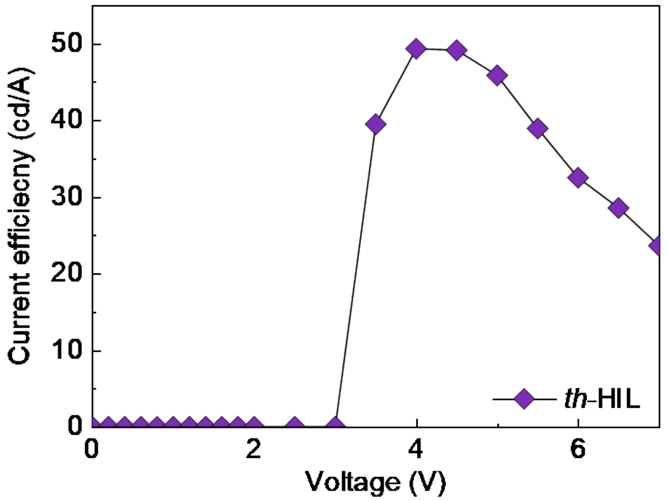

Fig. S38. Current efficiency versus voltage characteristics of SOLEDs using *th*-HIL.

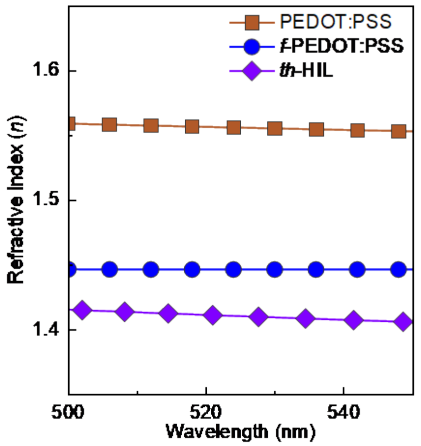

Fig. S39. Refractive index according to wavelength of PEDOT:PSS, *f*-PEDOT:PSS, and *th*-HIL.

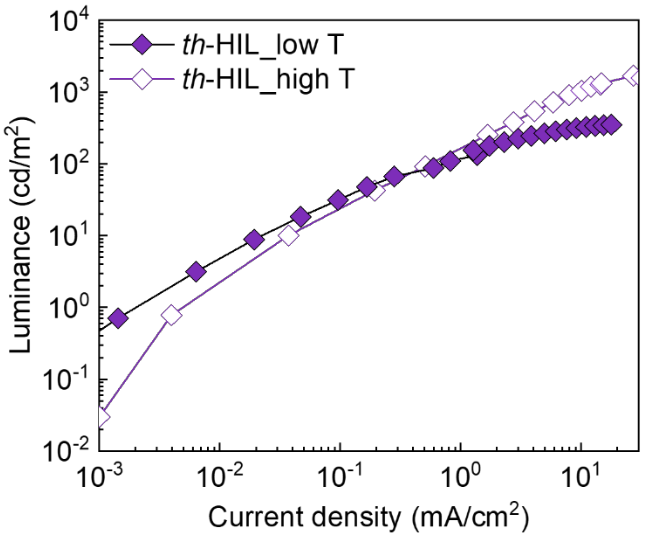

Fig. S40. Luminance versus current density characteristics of SOLEDs using *th*-HIL under low and high annealing temperatures.

We performed device optimization by systematically tuning the post-deposition annealing temperature of the polymer-containing EML and adjusting the PFSA ratio in the hole injection layer to enhance electrical conductivity. To achieve higher luminance operation, the hybrid EML thickness was increased from 40 nm to 50 nm, and the post-deposition annealing temperature was raised from 50 °C to 75 °C.

By increasing the post-deposition annealing temperature of hybrid EML, segmental ordering and better chain packing of the polymer backbone can be promoted, which enhanced electronic coupling with the small-molecule host matrix. This modification improved charge percolation pathways. These modifications enhance charge balance and carrier recombination volume, leading to increased luminance output. Concurrently, we adjusted the PFSA content in the HIL to reduce excessive electron blocking and improve electrical conductivity of itself. These modifications significantly improved charge transport in the hybrid EML, which resulted in devices with

increased current density, higher luminance, and substantially suppressed efficiency roll-off at a high luminance.

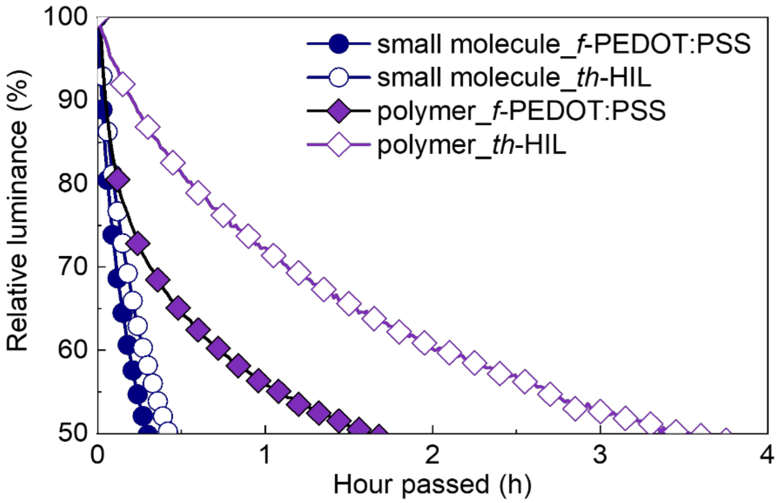

Fig. S41. Luminance decay of SOLEDs under constant current operation, comparing all-small-molecule EMLs (mCP:DBFPO:ν-DABNA) and polymer-sensitized EMLs (mCP:DBFPO:PDBA-SAF-P8P:ν-DABNA) with different hole injection layers.

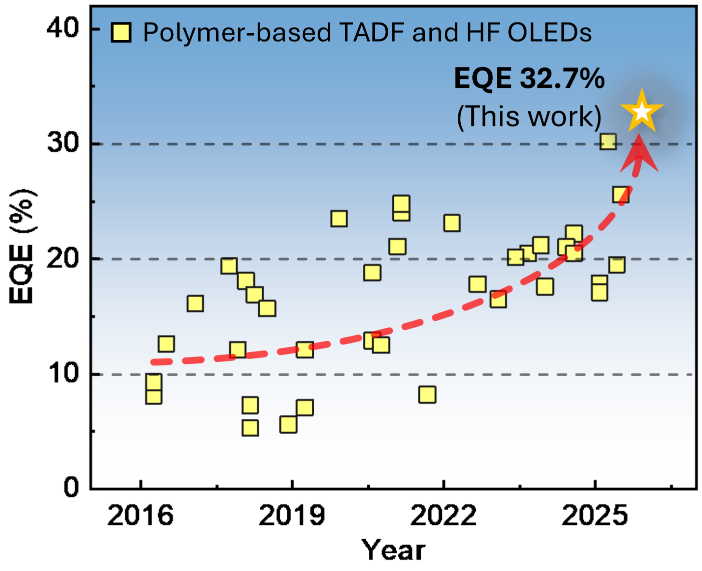

Fig. S42. Summary of the reported device efficiency for solution-processed polymer TADF or HF OLEDs across the entire visible spectrum.

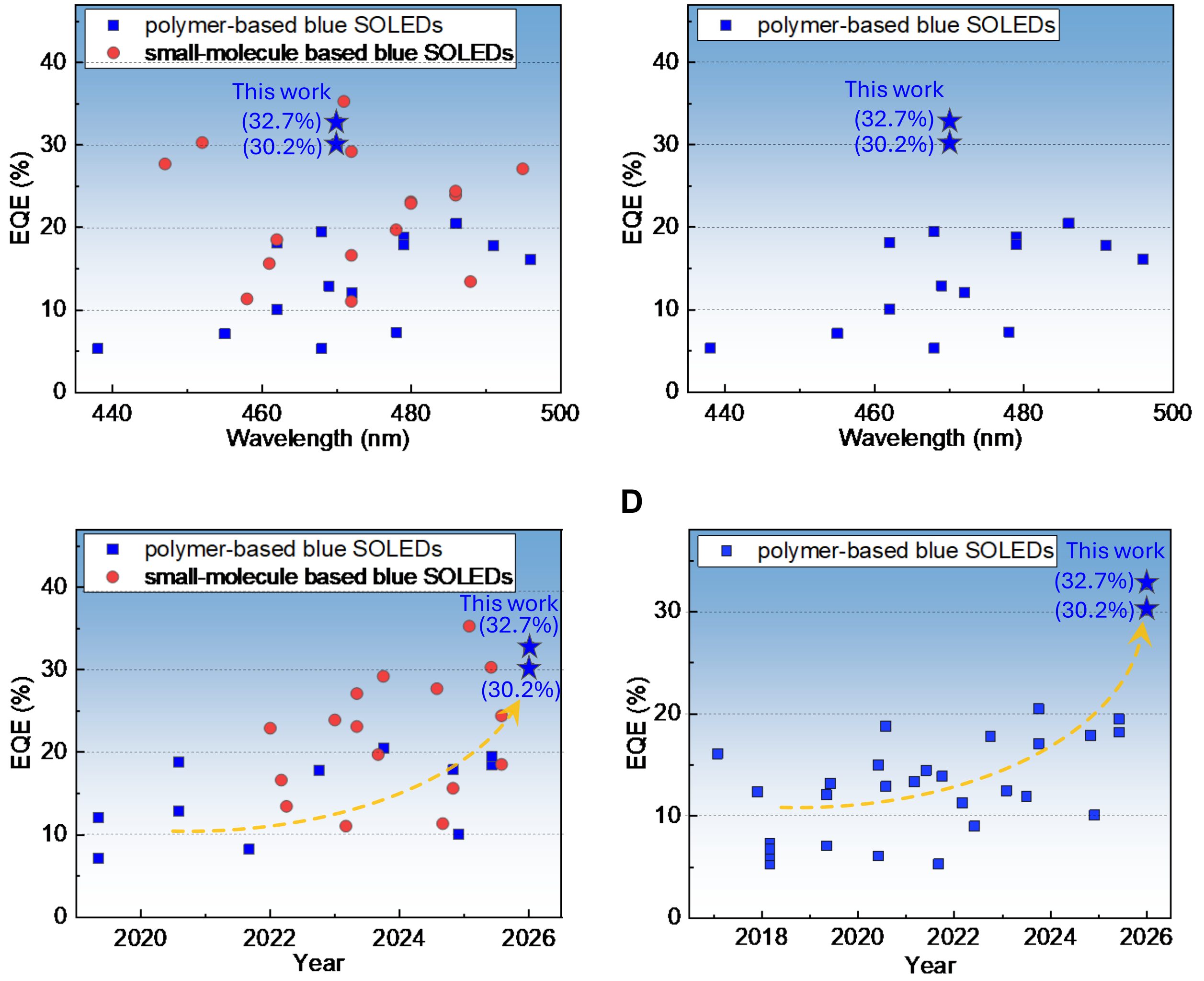

Fig. S43. Comparison of reported EQE of solution-processed blue OLEDs. (A) polymer-based and small-molecule-based devices, and (B) polymer-based devices as a function of emission wavelength, (C) polymer-based and small-molecule-based devices, and (D) polymer-based devices as a function of publication year.

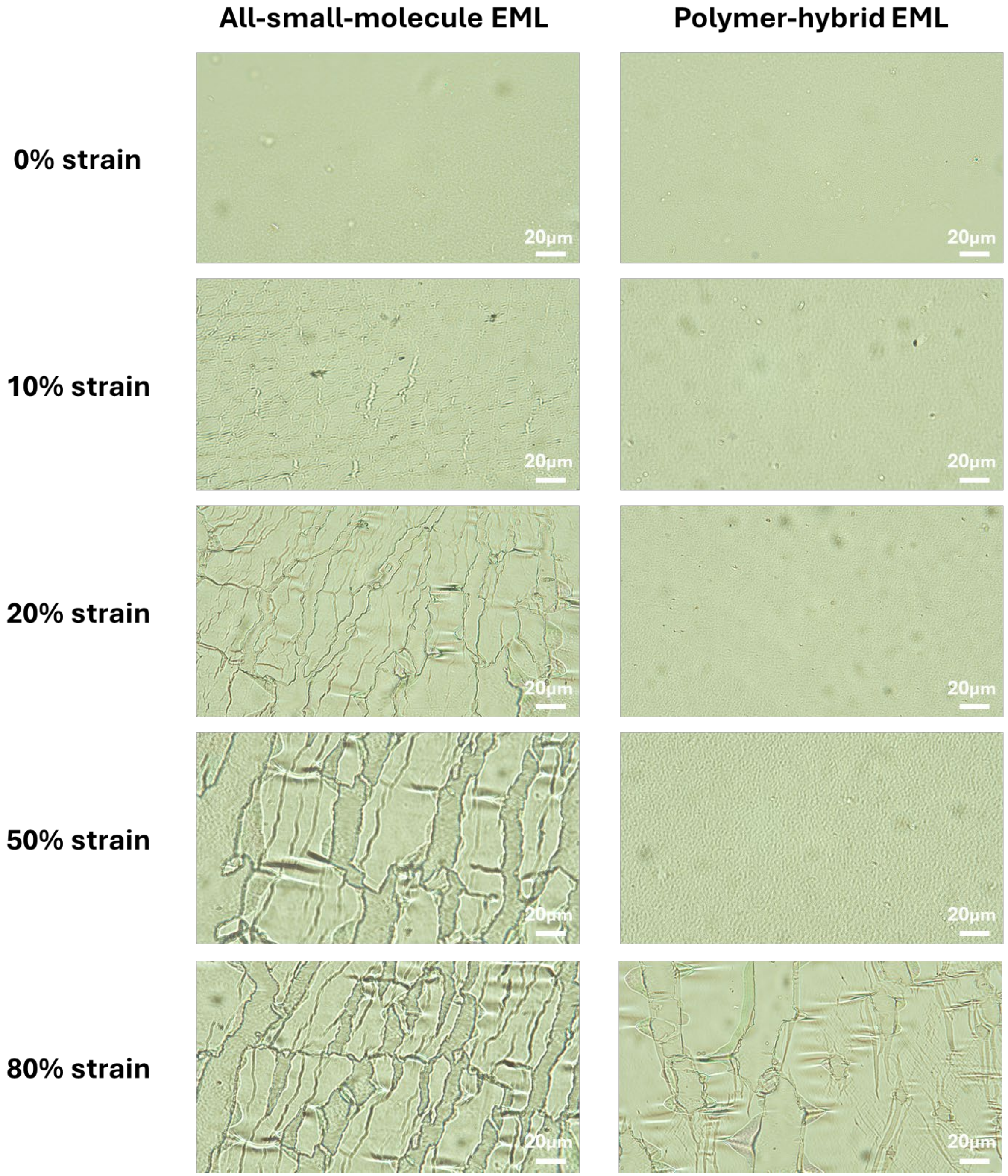

Fig. S44. Optical microscopy images of EML films under tensile strain. All-small-molecule (mCP:DBFPO) and polymer-hybrid EML (mCP:DBFPO:PDBA-SAF-P8P) under increasing tensile strain (0%, 10%, 20%, 50%, and 80%).

All-small-molecule films generally exhibit limited mechanical tolerance, with crack onset occurring at strain levels as low as ~10%, reflecting their brittle nature. In contrast, the polymer hybrid EML in this work maintained structural integrity up to ~80% strain, with crack onset delayed until this high deformation threshold, clearly demonstrating its potential for flexible and stretchable OLED applications, an area that remains extremely challenging for all-small-molecule systems.

Table S3. Summary of device performance for polymer-based blue TADF or HF OLEDs.

Year	Type	HIL	EML	EQE_{max} [%]	λ_{EL} [nm]	FWHM [nm]	CIE (x,y)	Ref.
2017	HF	PEDOT:PSS	mCP: DMAC-DP-Cz: PCzDP-10	16.1	496	~100	0.24, 0.40	(*55*)
2017	TADF	PEDOT:PSS	P-Ac95-TRZ05	12.4	472	~80	0.176, 0.269	(*24*)
2018	TADF	PEDOT:PSS	PBD-10	7.3	478	~100	0..20, 0.29	(*56*)
2018	TADF	PEDOT:PSS	mCP: P0	5.3	438	~60	0.16, 0.10	(*57*)
2018	TADF	PEDOT:PSS	mCP: P1	6.1	481	~120	0.20, 0.28	(*57*)
2018	TADF	PEDOT:PSS	mCP: P2	6.8	497	~110	0.22, 0.35	(*57*)
2019	TADF	PEDOT:PSS	mCP: P(BOPACTRZ-BPA)	13.2	477	~100	0.18, 0.32	(*58*)
2019	TADF	PEDOT:PSS	P1-05	7.1	455	~75	0.17, 0.17	(*59*)
2019	TADF	PEDOT:PSS	P2-05	12.1	472	~80	0.18, 0.27	(*59*)
2020	TADF	PEDOT:PSS	PNB-Ac-TRZ-5	12.9	469	~80	0.19, 0.26	(*60*)
2020	TADF	PEDOT:PSS	PNB-TAc-TRZ-5	18.8	479	~100	0.20, 0.31	(*60*)
2020	TADF	PEDOT:PSS	PBO-H-10	6.1	455	~85	0.18, 0.20	(*61*)
2020	TADF	PEDOT:PSS	PBO-F-10	15	474	~100	0.16, 0.27	(*61*)
2021	HF	PEDOT:PSS	DMAC-DP-Cz:P5	14.5	492	~100	0.18, 0.34	(*62*)
2021	TADF	PEDOT:PSS	Acridan: PAc-BSS	13.1	461	31	0.16, 0.12	(*63*)
2021	TADF	PEDOT:PSS	mCP: PTDD-50	5.3	468	~90	0.17, 0.22	(*64*)
2021	TADF	PEDOT:PSS	mCP: (S,S)-pTpAcDPS	13.9	496	~80	-	(*25*)
2022	TADF	PEDOT:PSS	PDCDC	9	498	~110	0.23, 0.39	(*26*)
2022	TADF	PEDOT:PSS	P1-05	11.3	483	~90	0.20, 0.37	(*65*)
2022	TADF	PEDOT:PSS	mCP: PCzBN1	17.8	491	27	0.10, 0.43	(*66*)
2023	TADF	PEDOT:PSS	mCBP: Alt-P36	11.9	490	~90	-	(*29*)
2023	TADF	PEOT:PSS:PFI	mCP: poly(DOPAcDSCz-TMP)	12.5	486	~90	0.22, 0.43	(*67*)
2023	TADF	PEDOT:PSS	mCPCN: polyTD-2	17.1	484	~100	0.19, 0.35	(*28*)
2023	HF	PEDOT:PSS	mCPCN: DMAC-DPS: polyTD-2	20.5	486	~90	0.20, 0.35	(*28*)
2024	HF	PEDOT:PSS	3Cz2BN: PCzDBN3	17.9	479	28	0.11, 0.25	(*27*)
2024	HF	PEDOT:PSS	3Cz2BN: PCzPBN5	10.1	462	31	0.14, 0.12	(*68*)
2025	TADF	PEOT:PSS:PFI	PSiCAT5	18.2	462	~110	0.16, 0.21	(*69*)
2025	TADF	PEOT:PSS:PFI	PMSiCAT20	19.5	468	~60	0.16, 0.25	(*69*)
**2026**	**HF**	***th*-HIL**	**mCP: DBFPO: PDBA-SAF-P8P: v-DABNA**	**30.2**	**470**	**18**	**0.127, 0.140**	**This work**
**2026**	**HF**	***th*-HIL**	**mCP: DBFPO: PDBA-SAF-P8P: v-DABNA**	**32.7**	**470**	**25**	**0.131, 0.182**	**This work**

Table S4. Summary of device performance for polymer-based TADF or HF OLEDs.

Year	Type	HIL	EML	EQE_{max} [%]	λ_{EL} [nm]	FWHM [nm]	CIE (x,y)	Ref.
2016	TADF	PEDOT:PSS	PAPTC	12.6	521	~90	0.30, 0.59	(*70*)
2016	TADF	PEDOT:PSS	TCTA: TAPC: pCzBP	8.1	500	~130	0.28, 0.43	(*71*)
2016	TADF	PEDOT:PSS	TCTA: TAPC: pAzBP	9.3	548	~90	0.38, 0.57	(*71*)
2017	TADF	PEDOT:PSS	P-Ac95-TRZ05	12.1	472	~80	0.176, 0.269	(*24*)
2017	HF	PEDOT:PSS	mCP: DMAC-DP-Cz: PCzDP-10	16.1	496	~100	0.24, 0.40	(*55*)
2017	TADF	PEDOT:PSS	mCP: PFSOTT2	19.4	592	~130	0.51, 0.47	(*72*)
2018	TADF	PEDOT:PSS	PBD-10	7.3	478	~100	0.20, 0.29	(*56*)
2018	TADF	PEDOT:PSS	mCP: P0	5.3	438	~60	0.16, 0.10	(*57*)
2018	TADF	PEDOT:PSS	PABPC5	18.1	~550	~90	0.40, 0.56	(*73*)
2018	TADF	PEDOT:PSS	PCzAPT10	16.9	~530	~110	0.36, 0.55	(*74*)
2018	TADF	PEDOT:PSS	TCTA: TAPC: Cop-10	15.7	578	~130	0.46, 0.59	(*75*)
2018	TADF	PEDOT:PSS	ROC8:PFDMPE	5.62	606	~110	0.57, 0.42	(*76*)
2018	TADF	PEDOT:PSS	mCP: PCzDPT10	2.7	622	~120	0.57, 0.40	(*77*)
2019	TADF	PEDOT:PSS	P1-05	7.1	455	~75	0.17, 0.17	(*59*)
2019	TADF	PEDOT:PSS	P2-05	12.1	472	~80	0.18, 0.27	(*59*)
2019	TADF	PEDOT:PSS:PFI	mCP: poly(AcBPCz-TMP)	23.5	507	~100	0.25, 0.52	(*78*)
2020	TADF	PEDOT:PSS	PNB-Ac-TRZ-5	12.9	469	~80	0.19, 0.26	(*60*)
2020	TADF	PEDOT:PSS	PNB-TAc-TRZ-5	18.8	479	~100	0.20, 0.31	(*60*)
2020	TADF	PEDOT:PSS	PCzAQC0.5	12.5	620	~110	0.56, 0.42	(*79*)
2021	TADF	PEDOT:PSS	mCP: PCzDD-50	8.2	488	~90	0.22, 0.35	(*64*)
2021	TADF	PEDOT:PSS:PFI	mCP: poly(TMTPA–DCB)	24	~540	~100	0.34, 0.57	(*80*)
2021	TADF	PEDOT:PSS	mCP: PCPTCN-1/4	21.1	519	~120	0.41, 0.52	(*81*)
2021	TADF	PEDOT:PSS:PFI	mCP: PSAQF10	24.8	608	~120	0.48, 0.49	(*82*)
2022	TADF	PEDOT:PSS	PCzBN1	17.8	491	27	0.10, 0.43	(*66*)
2022	TADF	PEDOT:PSS	pBP-PXZ:CBP	23.1	578	~90	0.49, 0.49	(*83*)
2023	TADF	PEDOT:PSS:PFI	mCP: poly(DOPAcBPCz-TMP)	16.5	519	~90	0.37, 0.57	(*67*)
2023	HF	PEDOT:PSS	mCPCN:DMAC-DPS: polyTD-2	20.5	486	~100	0.20, 0.35	(*28*)
2023	HF	PEDOT:PSS:PFI	PhtBuPAD: poly(TMTPA-DCB)	21.2	510	~90	0.32, 0.59	(*43*)
2023	TADF	PEDOT:PSS	mCP: p-PXZ-XN	17.6	572	~100	0.47, 0.52	(*84*)
2023	TADF	PEDOT:PSS	mCP-CN: Pnai3705	20.2	610	~110	0.57, 0.38	(*85*)
2023	TADF	PEDOT:PSS	R-P	6.2	662	~140	0.63, 0.37	(*86*)
2024	HF	PEDOT:PSS	3Cz2BN: PCzPBN5	10.1	462	31	0.14, 0.12	(*68*)
2024	TADF	PEDOT:PSS	SimCP2:PCzTBN5	22.2	511	44	0.18, 0.62	(*87*)
2024	HF	PEDOT:PSS	mCP: P10	21	548	~110	0.39, 0.54	(*88*)
2024	TADF	PEDOT:PSS	mCP: p-PXZ-XN-Py	20.5	568	~120	0.46, 0.53	(*89*)
2025	TADF	PEDOT:PSS:PFI	PMSiCAT20	19.5	468	~60	0.16, 0.25	(*69*)

2025	HF	PEDOT:PSS	3Cz2BN:PCzDBN3	17.9	479	28	0.11, 0.25	(*27*)
2025	HF	PEDOT:PSS	mCP: 5cz-trz: PSi6	30.2	506	44	0.19, 0.61	(*16*)
2025	TADF	PEDOT:PSS	mCP: p-2PXZ-XN	25.6	568	~100	0.46, 0.52	(*90*)
2025	TADF	PEDOT:PSS	mCP: pDBT3705	17.1	596	89	0.55, 0.44	(*91*)
2025	TADF	PEDOT:PSS	mCP-CN: pNAI-DBF3705	10	616	~100	0.59, 0.39	(*92*)
2025	TADF	PEDOT:PSS	PNAI-AcCz	2.6	659	~110	0.67, 0.33	(*93*)
**2026**	**HF**	***th*-HIL**	**mCP: DBFPO: PDBA-SAF-P8P: v-DABNA**	**30.2**	**470**	**18**	**0.127, 0.140**	**This work**
**2026**	**HF**	***th*-HIL**	**mCP: DBFPO: PDBA-SAF-P8P: v-DABNA**	**32.7**	**470**	**25**	**0.131, 0.182**	**This work**

Table S5. Summary of device performance for solution-processed blue TADF or HF OLEDs.

Year	Material type	Emission type	HIL	EML	EQE_{max} [%]	$λ_{EL}$ [nm]	FWHM [nm]	CIE (x,y)	Ref.
2017	Polymer	HF	PEDOT:PSS	mCP: DMAC-DP-Cz: PCzDP-10	16.1	496	~100	0.24, 0.40	(*55*)
2018	Polymer	TADF	PEDOT:PSS	PBD-10	7.3	478	~100	0..20, 0.29	(*56*)
2018	Polymer	TADF	PEDOT:PSS	mCP: P0	5.3	438	~60	0.16, 0.10	(*57*)
2019	Polymer	TADF	PEDOT:PSS	P1-05	7.1	455	~75	0.17, 0.17	(*59*)
2019	Polymer	TADF	PEDOT:PSS	P2-05	12.1	472	~80	0.18, 0.27	(*59*)
2020	Polymer	TADF	PEDOT:PSS	PNB-Ac-TRZ-5	12.9	469	~80	0.19, 0.26	(*60*)
2020	Polymer	TADF	PEDOT:PSS	PNB-TAc-TRZ-5	18.8	479	~100	0.20, 0.31	(*60*)
2021	Small-molecule	TADF	Plexcore OC AQ-1200	Polymer C: V-DABNA-Mes	22.9	480	27	0.09, 0.21	(*94*)
2021	Polymer	TADF	PEDOT:PSS	mCP: PTDD-50	5.3	468	~90	0.17, 0.22	(*64*)
2022	Small-molecule	HF	PEDOT:PSS	PYD2-DBN: Au-1: v-DABNA	16.6	472	23	0.14, 0.18	(*95*)
2022	Small-molecule	TADF	PEDOT:PSS	mCP: BON-D1	13.4	488	39	0.13, 0.44	(*96*)
2022	Small-molecule	HF	PEDOT:PSS	mCP: 5tBuCzTRZ: DtBuCzB	23.9	486	28	0.12, 0.38	(*97*)
2022	Polymer	TADF	PEDOT:PSS	mCP: PCzBN1	17.8	491	27	0.10, 0.43	(*66*)
2023	Small-molecule	TADF	PEDOT:PSS	mCP: TBN-TPA	11	472	~30	0.12, 0.16	(*98*)
2023	Small-molecule	HF	PEDOT:PSS	DMBN-PTC: 5TCzBN:2TCzBN	23.1	480	36	0.14, 0.34	(*50*)
2023	Small-molecule	HF	PEDOT:PSS	DMIC-TRZ: BN-36Cz-BN	27.1	495	31	0.11, 0.50	(*99*)
2023	Small-molecule	TADF	PEDOT:PSS	mCP: BSeN-TCZ	19.7	478	31	0.11, 0.21	(*100*)
2023	Small-molecule	HF	PEDOT:PSS	mCP: 5CzTRZ: t-BuCz-DABNA	29.2	472	16.6	0.139, 0.189	(*22*)
2023	Polymer	HF	PEDOT:PSS	mCPCN: DMAC-DPS: polyTD-2	20.5	486	~90	0.20, 0.35	(*28*)
2024	Small-molecule	TADF	PEDOT:PSS	mCPCN: Me-FOBN	11.3	458	32	0.14, 0.09	(*101*)
2024	Small-molecule	HF	PEDOT:PSS	mCP: 5tCzPPm: BSS-TBCz	15.6	461	36	0.14, 0.13	(*102*)
2024	Small-molecule	HF	PEDOT:PSS	mCP: TB-tCz: DOBN	27.7	447	20	0.15, 0.03	(*103*)
2024	Polymer	HF	PEDOT:PSS	3Cz2BN: PCzDBN3	17.9	479	28	0.11, 0.25	(*27*)
2024	Polymer	HF	PEDOT:PSS	3Cz2BN: PCzPBN5	10.1	462	31	0.14, 0.12	(*68*)
2025	Small-molecule	HF	PEDOT:PSS	8CzTPS: 5CzTRZ: D2-DBN	35.3	471	17	0.137, 0.176	(*49*)
2025	Small-molecule	TADF	PEDOT:PSS	mCPBC: tBO-4B	30.3	452	16	0.147, 0.042	(*51*)
2025	Small-molecule	TADF	PEDOT:PSS	mCP: TRZCz	24.4	486	79	0.20, 0.39	(*104*)

2025	Small-molecule	HF	PEDOT:PSS	mCP: TRZCz: BSS-TBCz	18.5	462	43	0.16, 0.19	(*104*)
2025	Polymer	TADF	PEOT:PSS:PFI	PSiCAT5	18.2	462	~110	0.16, 0.21	(*69*)
2025	Polymer	TADF	PEOT:PSS:PFI	PMSiCAT20	19.5	468	~60	0.16, 0.25	(*69*)
**2026**	**Polymer**	**HF**	***th*-HIL**	**mCP: DBFPO: PDBA-SAF-P8P: v-DABNA**	**30.2**	**470**	**18**	**0.127, 0.140**	**This work**
**2026**	**Polymer**	**HF**	***th*-HIL**	**mCP: DBFPO: PDBA-SAF-P8P: v-DABNA**	**32.7**	**470**	**25**	**0.131, 0.182**	**This work**

REFERENCES

1. S. Sudheendran Swayamprabha, D. Kumar Dubey, R. A. K. Yadav, M. R. Nagar, A. Sharma, F.-C. Tung, J.-H. Jou, Approaches for long lifetime organic light emitting diodes. *Adv. Sci.* **8**, 2002254 (2021).

2. H. W. Chen, J. H. Lee, B. Y. Lin, S. Chen, S. T. Wu, Liquid crystal display and organic light-emitting diode display: Present status and future perspectives. *Light Sci. Appl.* **7**, 17168 (2018).

3. S. J. Zou, Y. Shen, F. M. Xie, J. De Chen, Y. Q. Li, J. X. Tang, Recent advances in organic light-emitting diodes: Toward smart lighting and displays. *Mater. Chem. Front.* **4**, 788–820 (2020).

4. D. Park, Y. J. Kim, Y. K. Park, 79-2: Hyperrealism in full ultra high-definition 8K display. *SID Symp. Dig. Tech. Pap.* **50**, 1138–1141 (2019).

5. Y. Yin, M. U. Ali, W. Xie, H. Yang, H. Meng, Evolution of white organic light-emitting devices: From academic research to lighting and display applications. *Mater. Chem. Front.* **3**, 970–1031 (2019).

6. J. Y. Woo, M.-H. Park, S.-H. Jeong, Y.-H. Kim, B. Kim, T.-W. Lee, T.-H. Han, Advances in solution-processed OLEDs and their prospects for use in displays. *Adv. Mater.* **35**, e2207454 (2023).

7. S. Wang, H. Zhang, B. Zhang, Z. Xie, W. Y. Wong, Towards high-power-efficiency solution-processed OLEDs: Material and device perspectives. *Mater. Sci. Eng. R Rep.* **140**, 100547 (2020).

8. H. Nakanotani, T. Higuchi, T. Furukawa, K. Masui, K. Morimoto, M. Numata, H. Tanaka, Y. Sagara, T. Yasuda, C. Adachi, High-efficiency organic light-emitting diodes with fluorescent emitters. *Nat. Commun.* **5**, 4016 (2014).

9. T. Furukawa, H. Nakanotani, M. Inoue, C. Adachi, Dual enhancement of electroluminescence efficiency and operational stability by rapid upconversion of triplet excitons in OLEDs. *Sci. Rep.* **5**, 8429 (2015).

10. C. Y. Chan, M. Tanaka, Y. T. Lee, Y. W. Wong, H. Nakanotani, T. Hatakeyama, C. Adachi, Stable pure-blue hyperfluorescence organic light-emitting diodes with high-efficiency and narrow emission. *Nat. Photonics* **15**, 203–207 (2021).

11. K. Stavrou, L. G. Franca, A. Danos, A. P. Monkman, Key requirements for ultraefficient sensitization in hyperfluorescence organic light-emitting diodes. *Nat. Photonics* **18**, 554–561 (2024).

12. H. S. Kim, H. J. Cheon, D. Lee, W. Lee, J. Kim, Y.-H. Kim, S. Yoo, Toward highly efficient deep-blue OLEDs: Tailoring the multiresonance-induced TADF molecules for suppressed excimer formation and near-unity horizontal dipole ratio. *Sci. Adv.* **9**, eadf1388 (2023).

13. H. Liu, Y. Fu, J. Chen, B. Z. Tang, Z. Zhao, Energy-efficient stable hyperfluorescence organic light-emitting diodes with improved color purities and ultrahigh power efficiencies based on low-polar sensitizing systems. *Adv. Mater.* **35**, e2212237 (2023).

14. H. Lee, R. Braveenth, S. Muruganantham, C. Y. Jeon, H. S. Lee, J. H. Kwon, Efficient pure blue hyperfluorescence devices utilizing quadrupolar donor-acceptor-donor type of thermally activated delayed fluorescence sensitizers. *Nat. Commun.* **14**, 419 (2023).

15. D. Zhang, X. Song, A. J. Gillett, B. H. Drummond, S. T. E. Jones, G. Li, H. He, M. Cai, D. Credgington, L. Duan, Efficient and stable deep-blue fluorescent organic light-emitting diodes employing a sensitizer with fast triplet upconversion. *Adv. Mater.* **32**, 1908355 (2020).

16. L. Hua, H. Wu, Z. Xia, M. Li, Y. Liu, S. Yan, W. Zhu, J. Y. Lee, Z. Ren, Y. Wang, Narrowband emissive solution-processed polymer organic light-emitting diodes with external quantum efficiency above 30%. *Adv. Mater.* **37**, 2502180 (2025).

17. D. Zhang, L. Duan, C. Li, Y. Li, H. Li, D. Zhang, Y. Qiu, High-efficiency fluorescent organic light-emitting devices using sensitizing hosts with a small singlet–triplet exchange energy. *Adv. Mater.* **26**, 5050–5055 (2014).

18. S. Nam, J. W. Kim, H. J. Bae, Y. M. Maruyama, D. Jeong, J. Kim, J. S. Kim, W. J. Son, H. Jeong, J. Lee, S. G. Ihn, H. Choi, Improved efficiency and lifetime of deep-blue hyperfluorescent

organic light-emitting diode using Pt(II) complex as phosphorescent sensitizer. *Adv. Sci.* **8**, e2100586 (2021).

19. K. R. Naveen, H. Lee, R. Braveenth, D. Karthik, K. J. Yang, S. J. Hwang, J. H. Kwon, Achieving high efficiency and pure blue color in hyperfluorescence organic light emitting diodes using organo-boron based emitters. *Adv. Funct. Mater.* **32**, 2110356 (2022).

20. E. Cha, J. Jeon, H. W. Kim, H. U. Lee, J. Y. Woo, J. S. Yeo, H. B. Kwon, S. B. Cho, T.-H. Han, Self-reinforcing degradation of solution-processed small-molecule OLEDs: Excited-states and molecular interactions as key triggers. *Adv. Funct. Mater.* **35**, e08146 (2025).

21. K. Stavrou, A. Danos, T. Hama, T. Hatakeyama, A. Monkman, Hot vibrational states in a high-performance multiple resonance emitter and the effect of excimer quenching on organic light-emitting diodes. *ACS Appl. Mater. Interfaces* **13**, 8643–8655 (2021).

22. K. Zhang, X. Wang, Y. Chang, Y. Wu, S. Wang, L. Wang, Carbazole-decorated organoboron emitters with low-lying HOMO levels for solution-processed narrowband blue hyperfluorescence OLED devices. *Angew. Chem. Int. Ed. Engl.* **62**, e202313084 (2023).

23. Q. Wei, Z. Ge, B. Voit, Thermally activated delayed fluorescent polymers: Structures, properties, and applications in OLED devices. *Macromol. Rapid Commun.* **40**, e1800570 (2019).

24. S. Shao, J. Hu, X. Wang, L. Wang, X. Jing, F. Wang, Blue thermally activated delayed fluorescence polymers with nonconjugated backbone and through-space charge transfer effect. *J. Am. Chem. Soc.* **139**, 17739–17742 (2017).

25. Y. F. Wang, M. Li, J. M. Teng, H. Y. Zhou, W. L. Zhao, C. F. Chen, Chiral TADF-active polymers for high-efficiency circularly polarized organic light-emitting diodes. *Angew. Chem. Int. Ed. Engl.* **60**, 23619–23624 (2021).

26. C. Li, A. K. Harrison, Y. Liu, Z. Zhao, F. B. Dias, C. Zeng, S. Yan, M. R. Bryce, Z. Ren, TADF dendronized polymer with vibrationally enhanced direct spin-flip between charge-transfer states for efficient non-doped solution-processed OLEDs. *Chem. Eng. J.* **435**, 134924 (2022).

27. W. Luo, T. Wang, Z. Huang, H. Huang, N. Li, C. Yang, Blue TADF conjugated polymers with multi-resonance feature toward solution-processable narrowband blue OLEDs. *Adv. Funct. Mater.* **34**, 2310042 (2024).

28. Y. Liu, Y. Xie, L. Hua, S. Li, X. Tong, S. Ying, S. Yan, Z. Ren, High-efficiency TADF polymers with a spatially confined conjugated backbone enable solution-processable blue OLEDs realizing over 20% EQE. *Adv. Opt. Mater.* **12**, 2301811 (2024).

29. X. Tong, Z. Zhao, L. Hua, Y. Zhang, B. Xu, Y. Liu, S. Yan, Z. Ren, Modulating backbone conjugation of polymeric thermally activated delayed fluorescence emitters for high-efficiency blue OLEDs. *Adv. Funct. Mater.* **33**, 2305324 (2023).

30. H. Lim, H. J. Cheon, S. J. Woo, S. K. Kwon, Y. H. Kim, J. J. Kim, Highly efficient deep-blue OLEDs using a TADF emitter with a narrow emission spectrum and high horizontal emitting dipole ratio. *Adv. Mater.* **32**, e2004083 (2020).

31. T.-H. Han, M.-R. Choi, C.-W. Jeon, Y.-H. Kim, S.-K. Kwon, T.-W. Lee, Ultrahigh-efficiency solution-processed simplified small-molecule organic light-emitting diodes using universal host materials. *Sci. Adv.* **2**, e1601428 (2016).

32. Y.-H. Kim, T.-H. Han, C. Lee, Y.-H. Kim, Y. Yang, T.-W. Lee, Molecular-scale strategies to achieve high efficiency and low efficiency roll-off in simplified solution-processed organic light-emitting diodes. *Adv. Funct. Mater.* **30**, 2005292 (2020).

33. Y.-H. Kim, C. Wolf, H. Cho, S.-H. Jeong, T.-W. Lee, Highly efficient, simplified, solution-processed thermally activated delayed-fluorescence organic light-emitting diodes. *Adv. Mater.* **28**, 734–741 (2016).

34. T.-H. Han, M.-R. Choi, S.-H. Woo, S.-Y. Min, C.-L. Lee, T.-W. Lee, Molecularly controlled interfacial layer strategy toward highly efficient simple-structured organic light-emitting diodes. *Adv. Mater.* **24**, 1487–1493 (2012).

35. Y. J. Cho, K. S. Yook, J. Y. Lee, High efficiency in a solution-processed thermally activated delayed-fluorescence device using a delayed-fluorescence emitting material with improved solubility. *Adv. Mater.* **26**, 6642–6646 (2014).

36. A. S. D. Sandanayaka, K. Yoshida, T. Matsushima, C. Adachi, Exciton quenching behavior of thermally activated delayed fluorescence molecules by charge carriers. *J. Phys. Chem. C* **119**, 7631–7636 (2015).

37. Y. Li, J. Ding, C. Liang, X. Zhang, J. Zhang, D. S. Jakob, B. Wang, X. Li, H. Zhang, L. Li, Y. Yang, G. Zhang, X. Zhang, W. Du, X. Liu, Y. Zhang, Y. Zhang, X. Xu, X. Qiu, H. Zhou, Nanoscale heterogeneous distribution of surface energy at interlayers in organic bulk-heterojunction solar cells. *Joule* **5**, 3154–3168 (2021).

38. I. D. Parker, Carrier tunneling and device characteristics in polymer light-emitting diodes. *J. Appl. Phys.* **75**, 1656–1666 (1994).

39. M. Gao, P. L. Burn, A. Pivrikas, Balanced hole and electron transport in Ir(ppy)3:TCTA blends. *ACS Photonics* **8**, 2425–2430 (2021).

40. M. Singh, J. H. Jou, S. Sahoo, S. Sujith, Z. K. He, G. Krucaite, S. Grigalevicius, C. W. Wang, High light-quality OLEDs with a wet-processed single emissive layer. *Sci. Rep.* **8**, 7133 (2018).

41. Z. Liu, W. Y. Zheng, P. Wei, Z. Xu, D. Song, B. Qiao, S. Zhao, The improved performance and mechanism of solution-processed blue PhOLEDs based on double electron transport layers. *RSC Adv.* **10**, 13215–13222 (2020).

42. J. Wang, J. Liu, S. Huang, X. Wu, X. Shi, C. Chen, Z. Ye, J. Lu, Y. Su, G. He, Y. Zheng, High efficiency green phosphorescent organic light-emitting diodes with a low roll-off at high brightness. *Org. Electron.* **14**, 2854–2858 (2013).

43. L. Yan, N. Su, Y. Yang, X. Li, J. Sun, S. Wang, L. Zhao, L. Ding, J. Ding, TADF polymer enables over 20% EQE in solution-processed green fluorescent OLEDs. *SmartMat* **5**, e1272 (2024).

44. D. H. Ahn, J. H. Jeong, J. Song, J. Y. Lee, J. H. Kwon, Highly efficient deep blue fluorescent organic light-emitting diodes boosted by thermally activated delayed fluorescence sensitization. *ACS Appl. Mater. Interfaces* **10**, 10246–10253 (2018).

45. G. W. Kim, H. W. Bae, R. Lampande, I. J. Ko, J. H. Park, C. Y. Lee, J. H. Kwon, Highly efficient single-stack hybrid cool white OLED utilizing blue thermally activated delayed fluorescent and yellow phosphorescent emitters. *Sci. Rep.* **8**, 16263 (2018).

46. T.-H. Han, W. Song, T.-W. Lee, Elucidating the crucial role of hole injection layer in degradation of organic light-emitting diodes. *ACS Appl. Mater. Interfaces* **7**, 3117–3125 (2015).

47. V. R. Nikitenko, V. I. Arkhipov, Y. H. Tak, J. Pommerehne, H. Bässler, H. H. Hörhold, The overshoot effect in transient electroluminescence from organic bilayer light emitting diodes: Experiment and theory. *J. Appl. Phys.* **81**, 7514–7525 (1997).

48. T.-H. Han, Y.-H. Kim, M.-H. Kim, W. Song, T.-W. Lee, Synergetic influences of mixed-host emitting layer structures and hole injection layers on efficiency and lifetime of simplified phosphorescent organic light-emitting diodes. *ACS Appl. Mater. Interfaces* **8**, 6152–6163 (2016).

49. K. Zhang, X. Wang, M. Wang, S. Wang, L. Wang, Solution-processed blue narrowband OLED devices with external quantum efficiency beyond 35% through horizontal dipole orientation induced by electrostatic interaction. *Angew. Chem. Int. Ed. Engl.* **64**, e202423812 (2025).

50. D. Liu, Y. He, W. Qiu, X. Peng, M. Li, D. Li, J. Pu, J. Yang, Y. Gan, G. Yang, G. Sun, C. Shen, X. Cai, S. J. Su, Management of host–guest triplet exciton distribution for stable, high-efficiency, low roll-off solution-processed blue organic light-emitting diodes by employing triplet-energy-mediated hosts. *Adv. Funct. Mater.* **33**, 2301327 (2023).

51. Y. C. Cheng, X. Tang, R. Walia, T. Y. Zhang, X. C. Fan, J. Yu, K. Wang, C. Adachi, X. K. Chen, X. H. Zhang, High-efficiency and high color purity solution-processable deep-blue OLEDs enabled by linearly fully fused acceptor-donor-acceptor molecular design. *Adv. Mater.* **37**, e2500010 (2025).

52. Y. Wu, G. Zhuang, S. Cui, Y. Zhou, J. Wang, Q. Huang, P. Du, Through-space p-delocalization in a conjugated macrocycle consisting of [2.2]paracyclophane. *Chem. Commun.* **55**, 14617–14620 (2019).

53. C. Y. Chan, M. Tanaka, H. Nakanotani, C. Adachi, Efficient and stable sky-blue delayed fluorescence organic light-emitting diodes with CIE y below 0.4. *Nat. Commun.* **9**, 5036 (2018).

54. L. Yan, B. Chen, D. Wang, N. Su, L. Zhao, S. Wang, J. Ding, Efficient solution-processed narrowband green-emitting organic light emitting diodes sensitized by a thermally activated delayed fluorescence polymer. *J. Mater. Chem. C* **12**, 16827–16833 (2024).

55. G. Xie, J. Luo, M. Huang, T. Chen, K. Wu, S. Gong, C. Yang, Inheriting the characteristics of TADF small molecule by side-chain engineering strategy to enable bluish-green polymers with high PLQYs up to 74% and external quantum efficiency over 16% in light-emitting diodes. *Adv. Mater.* **29**, 1604223 (2017).

56. X. Zeng, J. Luo, T. Zhou, T. Chen, X. Zhou, K. Wu, Y. Zou, G. Xie, S. Gong, C. Yang, Using ring-opening metathesis polymerization of norbornene to construct thermally activated delayed fluorescence polymers: High-efficiency blue polymer light-emitting diodes. *Macromolecules* **51**, 1598–1604 (2018).

57. Y. Hu, W. Cai, L. Ying, D. Chen, X. Yang, X. F. Jiang, S. Su, F. Huang, Y. Cao, Novel efficient blue and bluish-green light-emitting polymers with delayed fluorescence. *J. Mater. Chem. C* **6**, 2690–2695 (2018).

58. X. Liu, J. Rao, X. Li, S. Wang, J. Ding, L. Wang, Teaching an old poly(arylene ether) new tricks: Efficient blue thermally activated delayed fluorescence. *iScience* **15**, 147–155 (2019).

59. J. Hu, Q. Li, X. Wang, S. Shao, L. Wang, X. Jing, F. Wang, Developing through-space charge transfer polymers as a general approach to realize full-color and white emission with thermally activated delayed fluorescence. *Angew. Chem. Int. Ed. Engl.* **58**, 8405–8409 (2019).

60. Q. Li, J. Hu, J. Lv, X. Wang, S. Shao, L. Wang, X. Jing, F. Wang, Through-space charge-transfer polynorbornenes with fixed and controllable spatial alignment of donor and acceptor for

high-efficiency blue thermally activated delayed fluorescence. *Angew. Chem. Int. Ed. Engl.* **59**, 20174–20182 (2020).

61. F. Chen, J. Hu, X. Wang, S. Shao, L. Wang, X. Jing, F. Wang, Through-space charge transfer blue polymers containing acridan donor and oxygen-bridged triphenylboron acceptor for highly efficient solution-processed organic light-emitting diodes. *Sci. China Chem.* **63**, 1112–1120 (2020).

62. T. Chen, Z. Chen, F. Ni, G. Xie, C. Yang, Sky-blue thermally activated delayed fluorescence polymers by using a conjugation-confined poly(aryl ether) main chain. *Polym. Chem.* **12**, 2490–2497 (2021).

63. F. Chen, L. Zhao, X. Wang, Q. Yang, W. Li, H. Tian, S. Shao, L. Wang, X. Jing, F. Wang, Novel boron- and sulfur-doped polycyclic aromatic hydrocarbon as multiple resonance emitter for ultrapure blue thermally activated delayed fluorescence polymers. *Sci. China Chem.* **64**, 547–551 (2021).

64. Y. Liu, S. Yan, Z. Ren, π-Conjugated polymeric light emitting diodes with sky-blue emission by employing thermally activated delayed fluorescence mechanism. *Chem. Eng. J.* **417**, 128089 (2021).

65. J. Hu, Y. Chang, F. Chen, Q. Yang, S. Shao, L. Wang, Design, synthesis, and properties of polystyrene-based through-space charge transfer polymers: Effect of triplet energy level of electron donor moiety on delayed fluorescence and electroluminescence performance. *J. Polym. Sci.* **60**, 1855–1863 (2022).

66. T. Wang, Y. Zou, Z. Huang, N. Li, J. Miao, C. Yang, Narrowband emissive TADF conjugated polymers towards highly efficient solution-processible OLEDs. *Angew. Chem. Int. Ed. Engl.* **61**, e202211172 (2022).

67. S. Liu, Y. Tian, L. Yan, S. Wang, L. Zhao, H. Tian, J. Ding, L. Wang, Color tuning in thermally activated delayed fluorescence polymers with carbazole and tetramethylphenylene backbone. *Macromolecules* **56**, 876–882 (2023).

68. T. Wang, S. Wang, J. Dong, G. Chen, J. Liu, M. Huang, Z. Chen, Z. Huang, C. Yang, Blue multiresonance thermally activated delayed fluorescence conjugated polymers for solution-processable narrowband blue organic light-emitting diodes with high color-purity. *Macromolecules* **58**, 363–371 (2025).

69. J. Lei, M. Zheng, S. Li, K. Dou, Y. Wang, H. Hao, J. Zhang, X. Li, Y. Cheng, P. W. M. Blom, Q. Niu, Y. Ma, High-efficiency blue polymer light-emitting diodes with neat thermally activated delayed fluorescence polymer films. *Adv. Opt. Mater.* **13**, 2500753 (2025).

70. Y. Zhu, Y. Zhang, B. Yao, Y. Wang, Z. Zhang, H. Zhan, B. Zhang, Z. Xie, Y. Wang, Y. Cheng, Synthesis and electroluminescence of a conjugated polymer with thermally activated delayed fluorescence. *Macromolecules* **49**, 4373–4377 (2016).

71. S. Y. Lee, T. Yasuda, H. Komiyama, J. Lee, C. Adachi, Thermally activated delayed fluorescence polymers for efficient solution-processed organic light-emitting diodes. *Adv. Mater.* **28**, 4019–4024 (2016).

72. Y. Wang, Y. Zhu, G. Xie, H. Zhan, C. Yang, Y. Cheng, Bright white electroluminescence from a single polymer containing a thermally activated delayed fluorescence unit and a solution-processed orange OLED approaching 20% external quantum efficiency. *J. Mater. Chem. C* **5**, 10715–10720 (2017).

73. Y. Yang, S. Wang, Y. Zhu, Y. Wang, H. Zhan, Y. Cheng, Thermally activated delayed fluorescence conjugated polymers with backbone-donor/pendant-acceptor architecture for nondoped OLEDs with high external quantum efficiency and low roll-off. *Adv. Funct. Mater.* **28**, 1706916 (2018).

74. Y. Zhu, Y. Yang, Y. Wang, B. Yao, X. Lin, B. Zhang, H. Zhan, Z. Xie, Y. Cheng, Improving luminescent performances of thermally activated delayed fluorescence conjugated polymer by inhibiting the intra- and interchain quenching. *Adv. Opt. Mater.* **6**, 1701320 (2018).

75. Y. Liu, Y. Wang, C. Li, Z. Ren, D. Ma, S. Yan, Efficient thermally activated delayed fluorescence conjugated polymeric emitters with tunable nature of excited states regulated via carbazole derivatives for solution-processed OLEDs. *Macromolecules* **51**, 4615–4623 (2018).

76. Y. Yang, L. Zhao, S. Wang, J. Ding, L. Wang, Red-emitting thermally activated delayed fluorescence polymers with poly(fluorene-co-3,3′-dimethyl diphenyl ether) as the backbone. *Macromolecules* **51**, 9933–9942 (2018).

77. Y. Wang, Y. Zhu, G. Xie, Q. Xue, C. Tao, Y. Le, H. Zhan, Y. Cheng, Red thermally activated delayed fluorescence polymers containing 9H-thioxanthen-9-one-10,10-dioxide acceptor group as pendant or incorporated in backbone. *Org. Electron.* **59**, 406–413 (2018).

78. J. Rao, X. Liu, X. Li, L. Yang, L. Zhao, S. Wang, J. Ding, L. Wang, Bridging small molecules to conjugated polymers: Efficient thermally activated delayed fluorescence with a methyl-substituted phenylene linker. *Angew. Chem. Int. Ed. Engl.* **59**, 1320–1326 (2020).

79. T. Wang, K. Li, B. Yao, Y. Chen, H. Zhan, Z. Xie, G. Xie, X. Yi, Y. Cheng, Rigidity and polymerization amplified red thermally activated delayed fluorescence polymers for constructing red and single-emissive-layer white OLEDs. *Adv. Funct. Mater.* **30**, 2002493 (2020).

80. J. Rao, L. Yang, X. Li, L. Zhao, S. Wang, H. Tian, J. Ding, L. Wang, Sterically-locked donor–acceptor conjugated polymers showing efficient thermally activated delayed fluorescence. *Angew. Chem. Int. Ed. Engl.* **60**, 9635–9641 (2021).

81. Y. Long, X. Chen, H. Wu, Z. Zhou, S. Sriram Babu, M. Wu, J. Zhao, M. P. Aldred, S. Liu, X. Chen, Z. Chi, J. Xu, Y. Zhang, Rigid polyimides with thermally activated delayed fluorescence for polymer light-emitting diodes with high external quantum efficiency up to 21%. *Angew. Chem. Int. Ed. Engl.* **60**, 7220–7226 (2021).

82. T. Wang, B. Yao, K. Li, Y. Chen, H. Zhan, X. Yi, Z. Xie, Y. Cheng, Backbone-acceptor/pendant-donor strategy for efficient thermally activated delayed fluorescence conjugated polymers with external quantum efficiency close to 25% and emission peak at 608 nm. *Adv. Opt. Mater.* **9**, 2001981 (2021).

83. Z. Zhao, Y. Liu, L. Hua, S. Yan, Z. Ren, Activating energy transfer tunnels by tuning local electronegativity of conjugated polymeric backbone for high-efficiency OLEDs with low efficiency roll-off. *Adv. Funct. Mater.* **32**, 2200018 (2022).

84. Y. Guo, Z. Zhao, L. Hua, Y. Liu, B. Xu, Y. Zhang, S. Yan, Z. Ren, Adjusting the electron-withdrawing ability of acceptors in thermally activated delayed fluorescence conjugated polymers for high-performance OLEDs. *ACS Appl. Mater. Interfaces* **16**, 1225–1233 (2023).

85. L. Hua, Y. Liu, H. Zhao, S. Chen, Y. Zhang, S. Yan, Z. Ren, Constructing high-efficiency orange-red thermally activated delayed fluorescence polymers by excited state energy levels regulation via backbone engineering. *Adv. Funct. Mater.* **33**, 2303384 (2023).

86. J. M. Teng, C. F. Chen, Chiral TADF polymers realizing highly-efficient deep-red circularly polarized electroluminescence over 660 nm. *Adv. Opt. Mater.* **11**, 2300550 (2023).

87. T. Wang, Z. Huang, H. Zhang, J. Miao, C. Yang, Multi-resonance TADF conjugated polymers toward highly efficient solution-processible narrowband green OLEDs. *Adv. Funct. Mater.* **34**, 2408119 (2024).

88. S. Li, Y. Xie, Y. Yin, J. Chen, Y. Cao, S. Ying, Y. Liu, Z. Ren, S. Yan, Intramolecular sensitization assisting high-efficiency TADF conjugated polymers with accelerating exciton spin flip for solution-processed electroluminescent devices. *Macromolecules* **57**, 5253–5261 (2024).

89. Y. Guo, L. Hua, H. Zhao, Y. Liu, S. Yan, Z. Ren, Modulating the excited states of thermally activated delayed fluorescence conjugated polymers by introducing hydrogen bonding into the acceptor for high-performance OLEDs. *J. Phys. Chem. C* **128**, 11924–11932 (2024).

90. Y. Guo, J. Zhao, L. Chen, H. Zhao, S. Li, Y. Liu, S. Yan, Z. Ren, Constructing intramolecular locks in the backbones of TADF conjugated polymers for high-performance solution-processed OLEDs. *Small* **21**, 2502592 (2025).

91. M. U. Khan, L. Hua, Y. Liu, H. Zhao, Y. Guo, Y. Wang, S. Yan, Z. Ren, Suppressing efficiency roll-off of orange-red thermally activated delayed fluorescence polymer-based OLEDs via copolymerizing co-hosts with cascade energy levels. *J. Mater. Chem. C* **13**, 4523–4532 (2025).

92. M. U. Khan, L. Hua, Z. Cao, Y. Liu, S. Yan, Z. Ren, Backbone modulation of thermally activated delayed fluorescence polymers for efficient orange-red emission in solution-processed OLEDs. *J. Mater. Chem. C* **13**, 22606–22615 (2025).

93. C. Li, X. Li, X. Luo, B. Xu, M. R. Bryce, Z. Ren, S. Yan, Deep-red TADF dendronized polymer for efficient non-doped solution-processed OLEDs. *J. Mater. Chem. C* **13**, 15628–15633 (2025).

94. S. Oda, B. Kawakami, Y. Yamasaki, R. Matsumoto, M. Yoshioka, D. Fukushima, S. Nakatsuka, T. Hatakeyama, One-shot synthesis of expanded heterohelicene exhibiting narrowband thermally activated delayed fluorescence. *J. Am. Chem. Soc.* **144**, 106–112 (2021).

95. D. Zhou, S. Wu, G. Cheng, C. M. Che, A gold(III)–TADF emitter as a sensitizer for high-color-purity and efficient deep-blue solution-processed OLEDs. *J. Mater. Chem. C* **10**, 4590–4596 (2022).

96. J. Liu, L. Chen, X. Wang, Q. Yang, L. Zhao, C. Tong, S. Wang, S. Shao, L. Wang, Multiple resonance dendrimers containing boron, oxygen, nitrogen-doped polycyclic aromatic emitters for narrowband blue-emitting solution-processed OLEDs. *Macromol. Rapid Commun.* **43**, e2200079 (2022).

97. L. Chen, Y. Chang, H. Shu, Q. Li, S. Shi, S. Wang, L. Wang, Achieving efficient solution-processed blue narrowband emitting OLEDs with small efficiency roll-off by using a bulky TADF sensitizer with high reverse intersystem crossing rate. *Adv. Opt. Mater.* **11**, 2201898 (2023).

98. H. Je, M. J. Cho, N. Y. Kwon, S. H. Park, M. J. Kang, H. Il Baek, J. Youn, C. W. Han, D. H. Choi, Universal polymeric hole transporting material for solution-processable green and blue thermally activated delayed fluorescence OLEDs. *ACS Appl. Mater. Interfaces* **15**, 9792–9799 (2023).

99. T. Wang, X. Yin, X. Cao, C. Yang, A simple approach to solution-processible small-molecule multi-resonance TADF emitters for high-performance narrowband OLEDs. *Angew. Chem. Int. Ed. Engl.* **62**, e202301988 (2023).

100. L. Yang, P. Wang, K. Zhang, S. Wang, S. Shao, L. Wang, Multiple resonance thermally activated delayed fluorescence dendrimers containing selenium-doped polycyclic aromatic hydrocarbon emitters for solution-processed narrowband blue OLEDs. *Dye. Pigment.* **216**, 111371 (2023).

101. X. Song, S. Shen, B. He, S. Zou, A. A. Vaitusionak, S. V Kostjuk, Y. Wang, Y. Wang, Y. Zhang, Dual multiresonance core strategy enable efficient pure blue organic light-emitting devices based on fluorene linkages. *Adv. Opt. Mater.* **12**, 2401505 (2024).

102. S. Shi, X. Wang, L. Zhao, J. Lv, S. Shao, L. Wang, Thermally activated delayed fluorescence sensitizer with through-space charge transfer manipulated by atom distribution for solution-processed deep-blue multi-resonance OLEDs. *Adv. Opt. Mater.* **13**, 2402490 (2025).

103. J. Jin, M. Chen, H. Jiang, B. Zhang, Z. Xie, W. Y. Wong, Construction of hybrid long- and short-range charge transfer excitations for high-performance deep-blue electroluminescence with CIEy < 0.04 and EQE > 35%. *ACS Mater. Lett.* **6**, 3246–3253 (2024).

104. S. Shi, X. Wang, L. Zhao, J. Lv, S. Shao, L. Wang, High-energy-level TADF sensitizers based on twisted diphenyltriazine acceptors for deep-blue multi-resonance OLEDs. *Adv. Opt. Mater.* **13**, e01323 (2025).
